# TNF blockade uncouples toxicity from antitumor efficacy induced with CD40 chemoimmunotherapy

**DOI:** 10.1172/jci.insight.146314

**Published:** 2021-07-22

**Authors:** Meredith L. Stone, Jesse Lee, Veronica M. Herrera, Kathleen Graham, Jae W. Lee, Austin Huffman, Heather Coho, Evan Tooker, Max I. Myers, Michael Giannone, Yan Li, Thomas H. Buckingham, Kristen B. Long, Gregory L. Beatty

**Affiliations:** 1 Division of Hematology/Oncology, Department of Medicine, University of Pennsylvania, Philadelphia, Pennsylvania, USA.; 2 Department of Biology, Mansfield University, Mansfield, Pennsylvania, USA.

**Keywords:** Immunology, Cancer immunotherapy, Cytokines

## Abstract

Agonist CD40 antibodies are under clinical development in combination with chemotherapy as an approach to prime for antitumor T cell immunity. However, treatment with anti-CD40 is commonly accompanied by both systemic cytokine release and liver transaminase elevations, which together account for the most common dose-limiting toxicities. Moreover, anti-CD40 treatment increases the potential for chemotherapy-induced hepatotoxicity. Here, we report a mechanistic link between cytokine release and hepatotoxicity induced by anti-CD40 when combined with chemotherapy and show that toxicity can be suppressed without impairing therapeutic efficacy. We demonstrate in mice and humans that anti-CD40 triggers transient hepatotoxicity marked by increased serum transaminase levels. In doing so, anti-CD40 sensitizes the liver to drug-induced toxicity. Unexpectedly, this biology is not blocked by the depletion of multiple myeloid cell subsets, including macrophages, inflammatory monocytes, and granulocytes. Transcriptional profiling of the liver after anti-CD40 revealed activation of multiple cytokine pathways including TNF and IL-6. Neutralization of TNF, but not IL-6, prevented sensitization of the liver to hepatotoxicity induced with anti-CD40 in combination with chemotherapy without impacting antitumor efficacy. Our findings reveal a clinically feasible approach to mitigate toxicity without impairing efficacy in the use of agonist CD40 antibodies for cancer immunotherapy.

## Graphical abstract

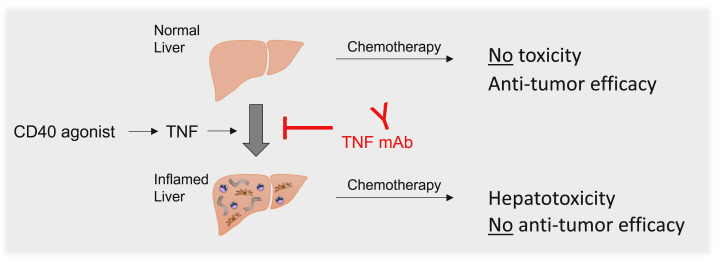

## Introduction

Most patients with solid cancers do not respond to T cell immunotherapies (1). However, preclinical modeling shows that myeloid agonists can enhance the therapeutic potential of T cell immunotherapy by triggering the activation of antigen-presenting cells (APCs) that are critical for the priming of antitumor T cell responses (2–5). In this regard, CD40 is a member of the TNF receptor superfamily and is expressed by APCs, including DCs. When ligated, CD40 “licenses” DCs with the capacity to prime T cells in an antigen-specific manner (6–8). In mouse models, anti-CD40 promotes T cell–dependent tumor regressions (9–11), particularly when combined with chemotherapy or immune checkpoint blockade (3–5, 12, 13). CD40 agonists also condition tumors for enhanced sensitivity to chemotherapy by modulating the extracellular matrix that surrounds tumor cells (14–16). Together, CD40 has emerged as a promising target for cancer immunotherapy.

Agonist CD40 antibodies have been under clinical development for more than a decade (2, 17). Clinical studies are actively investigating anti-CD40 in combination with chemotherapy, radiation, immune checkpoint blockade, and other immune modulatory agents (2, 17, 18). However, dose-limiting toxicities including cytokine release syndrome (CRS) and hepatotoxicity have hampered the development of agonist CD40 antibodies and remain a significant challenge for their translation to the clinic (16, 19, 20). Undoubtedly, insights into the determinants that underlie these immune-related toxicities will be important for maximizing the therapeutic potential and clinical success of agonist CD40 antibodies.

Systemic activation of the CD40 pathway invokes a cascade of immunological events characterized by an initial brisk release of cytokines (2, 14–16). During this process, myeloid cells and lymphocytes migrate to and become activated in lymphoid organs. Immune activation results in rapid onset of a transient immune-related hepatitis, during which the liver becomes hypersensitive to the toxic effects of chemotherapy (15, 21). We and others have shown that the delivery of chemotherapy within a few days after a CD40 agonist can be lethal in mice, illustrating the importance of carefully defined sequencing of immunotherapy with other drugs (15, 21, 22). However, the precise mechanism by which systemic activation of the CD40 pathway sensitizes the liver to drug toxicity remains undefined. In this study, we show that TNF released after treatment with anti-CD40 was responsible for sensitizing the liver to drug-induced hepatotoxicity but dispensable for therapeutic efficacy. In doing so, our results reveal a mechanistic link between CRS and hepatotoxicity induced by anti-CD40. Our findings also identify a feasible and easily translated approach using approved therapeutics to mitigate CD40-mediated immune-related toxicities.

## Results

### Treatment with an agonist CD40 antibody sensitizes the liver to lethal hepatotoxicity.

**Figure 1:**
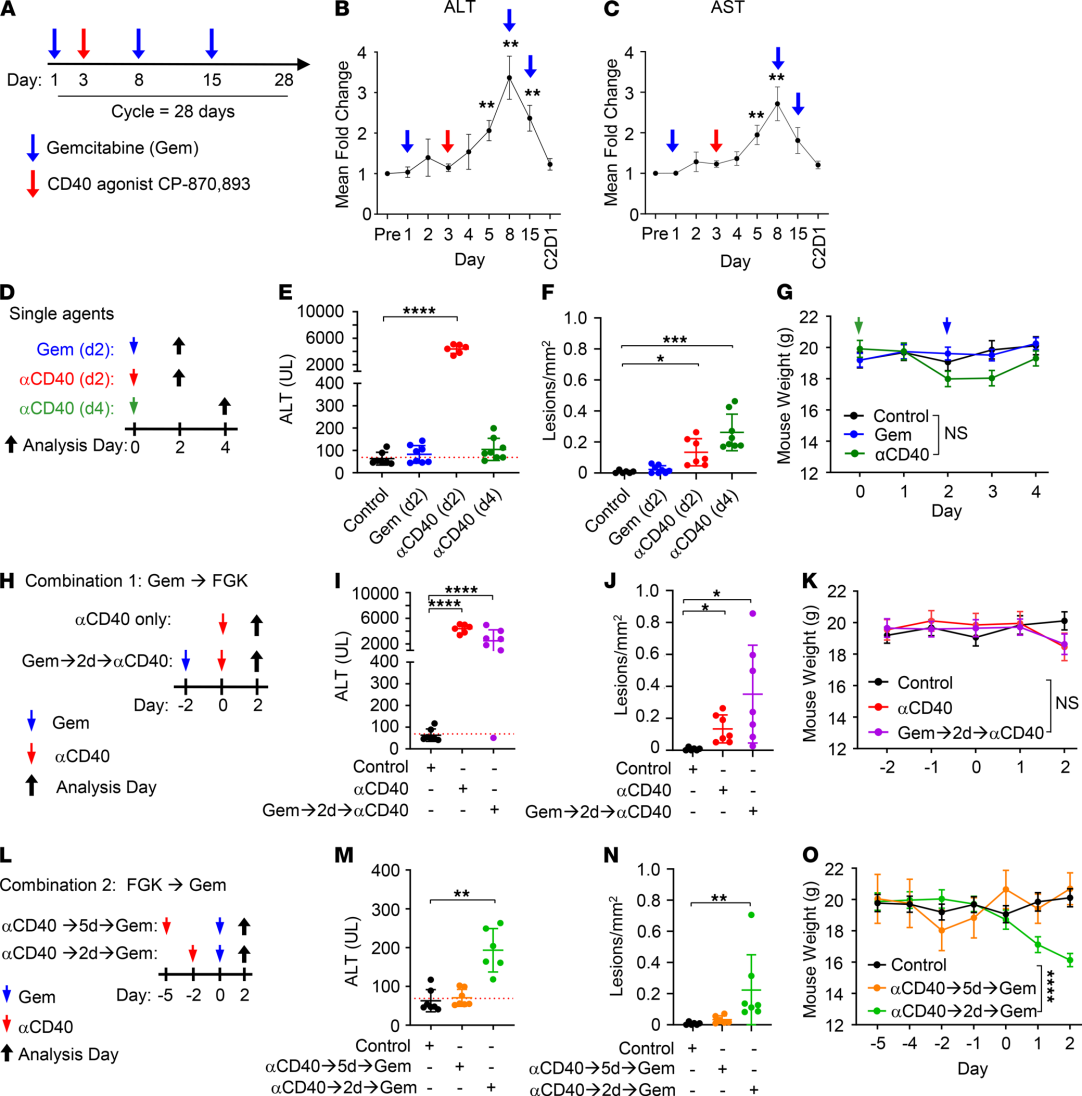
Systemic CD40 activation sensitizes the liver to chemotherapy-induced hepatotoxicity. (**A**) Treatment schema. Patients with chemotherapy-naive, surgically incurable PDAC received gemcitabine (1000 mg/m^2^) infused on days 1, 8, and 15 of each 28-day cycle, with CP-870,893 administered once on day 3 of each cycle. (**B**) ALT and (**C**) AST serum levels in patients treated as shown in **A**. *n* = 22 patients. One-way ANOVA with comparison to baseline (Pre) was performed. C2D1, cycle 2 day 1. (**D**) Study schema for **E**–**G**. Shown are (**E**) ALT serum levels and (**F**) number of lesions/mm^2^ in the liver detected on the day of analysis (shown in parentheses) after the indicated treatment. (**F**) Kruskal-Wallis with Dunn’s multiple comparisons test was performed. (**G**) Mouse weight over time after treatment (indicated by arrows). (**H**) Study schema for **I**–**K**. Shown are (**I**) ALT serum levels and (**J**) number of lesions/mm^2^ in the liver detected on day 2 after αCD40 treatment. (**J**) Brown-Forsythe and Welch’s 1-way ANOVA test with Dunnett’s T3 multiple-comparison test was performed. (**K**) Mouse weight over time. (**L**) Study schema for **M**–**O**. Shown are (**M**) ALT serum levels and (**N**) number of lesions/mm^2^ in the liver detected on day 2 after gemcitabine treatment. (**N**) Kruskal-Wallis with Dunn’s multiple-comparison test was performed. (**O**) Mouse weight over time. For **D**–**O**, *n* = 8 mice per group. Data are representative of ≥ 3 experimental replicates in control and αCD40→2d→Gem treated groups, ≥ 1 experimental replicate for all other groups. For **G**, **K**, and **O**, data shown are mean ± SEM with significance tested on day 2, and ordinary 1-way ANOVA with Dunnett’s multiple-comparison tests were performed. All other data shown are mean ± SD. For **E**, **I**, and **M**, red lines indicate upper range of the 95% CI for normal serum level of ALT derived from all experiments in the manuscript, and Kruskal-Wallis with Dunn’s multiple-comparison test was performed. Gem, gemcitabine; αCD40, clone FGK45; AST, aspartate aminotransferase; ALT, alanine aminotransferase. *P < 0.05; **P < 0.01; ***P < 0.001; ****P < 0.0001.

To investigate the biological effect of an agonist CD40 antibody on the liver, we first examined the kinetics of transaminase increases detected in the peripheral blood of patients after treatment with anti-CD40. To do this, we analyzed serum aspartate aminotransferase (AST) and alanine aminotransferase (ALT) levels collected on patients with pancreatic ductal adenocarcinoma (PDAC) treated on a clinical trial with the fully human agonist CD40 antibody CP-870,893 in combination with gemcitabine chemotherapy (16). In this study, patients received gemcitabine weekly on days 1, 8, and 15 with anti-CD40 administered on day 3 of each 28-day cycle (Figure 1A). As expected, we detected a small increase in AST and ALT within 24 hours after the first dose of gemcitabine chemotherapy, consistent with its known potential for hepatotoxicity. However, after anti-CD40 treatment on day 3, both AST and ALT levels increased significantly and within 48 hours had increased nearly 2-fold compared with pretreatment levels (Figure 1, B and C). Peak levels of AST and ALT were detected on day 8, which corresponded to 5 days after anti-CD40 treatment (Figure 1, B and C). Thereafter, the levels of AST and ALT declined despite weekly dosing of gemcitabine and returned to baseline levels by the beginning of day 1 of cycle 2.

We next analyzed the impact of anti-CD40 on the liver in mice. Consistent with our findings in patients, we found that the systemic administration of anti-CD40, but not gemcitabine, induced a cascade of events beginning with acute inflammation and hepatic damage, as seen by a transient serum transaminitis with increases in AST and ALT detected within 2 days after treatment (Figure 1, D and E, and Supplemental Figure 1A; supplemental material available online with this article; https://doi.org/10.1172/jci.insight.146314DS1). At this time point after anti-CD40, severe multifocal acute coagulative hepatocellular necrosis with occlusive and nonocclusive fibrin thrombi were detected in the liver (Figure 1F and Supplemental Figure 1B). These lesions were surrounded by macrophages and an atypical lymphocytic infiltrate and remained detectable in the liver 4 days after treatment (Figure 1F and Supplemental Figure 1, B and C). We next analyzed other organs, including the heart, lung, kidney, small intestine, large intestine, pancreas, and spleen, of mice 2 days after anti-CD40 (Supplemental Figure 1D). We found no overt pathology in the heart. In addition, only an increase in circulating myeloid and myeloid precursor cells without tissue damage was seen in other nonlymphoid organs. The spleen is characterized by white pulp hyperplasia consistent with the known role of CD40 in germinal center and memory B cell formation (23). We also examined mice longitudinally for changes in weight and found that a mild less than 10% decrease in weight loss was detected within 2 days after anti-CD40, with recovery observed 4 days after treatment (Figure 1G). In summary, anti-CD40 triggered mild but notable hepatotoxicity detected in both mice and humans within 2 days of treatment.

We and others have previously shown that the timing of chemotherapy administration relative to anti-CD40 is a critical determinant of the safety and tolerability of this treatment combination (15, 21, 22). To understand how gemcitabine chemotherapy might affect hepatotoxicity induced by anti-CD40, we compared the effect of administering gemcitabine prior to and after anti-CD40. In patients, chemotherapy is generally administered beginning 2 days prior to the delivery of anti-CD40 (16, 20). This timing is based on the hypothesis that chemotherapy elicits antigen release, allowing for uptake by APCs (2). Anti-CD40 is then administered with the intent to activate antigen-loaded APC and to trigger priming of tumor-specific T cells. However, 5 days after anti-CD40, chemotherapy is readministered based on standard-of-care dosing. We first tested gemcitabine given prior to anti-CD40 and found that gemcitabine administered at this time point did not affect CD40-induced serum transaminitis, hepatic lesion formation, or weight loss (Figure 1, H–K; and Supplemental Figure 2, A–C). This finding is consistent with a previous clinical study showing safety of a CD40 agonist when administered either 2 or 7 days after chemotherapy (24). In contrast, although serum AST and ALT levels were found to resolve to baseline 4 days after anti-CD40 alone (Figure 1E), treatment with gemcitabine 2 days after anti-CD40 caused transaminase levels to remain elevated with hepatic lesions seen on histological examination (Figure 1, L–N; and Supplemental Figure 2, A and B). Moreover, treatment with gemcitabine 2 days after anti-CD40 caused progressive weight loss, reaching less than 20% 2 days after gemcitabine administration, which required euthanasia per IACUC guidelines (Figure 1O and Supplemental Figure 2C). Examination of livers from mice treated with anti-CD40 and then subsequent chemotherapy revealed severe diffuse hepatic lipidosis (Supplemental Figure 2D). Similar results were observed with administering nab-paclitaxel chemotherapy 2 days after anti-CD40 (Supplemental Figure 2, E and F). Moreover, we found that the combination of gemcitabine and nab-paclitaxel, a standard-of-care regimen for pancreatic cancer that has been combined with anti-CD40 in patients (20), also produced toxicity when administered at 2 days after anti-CD40 treatment. However, this toxicity was not apparent when chemotherapy was delayed to 5 days after anti-CD40 (Figure 1, L–O; and Supplemental Figure 2, E and F). Together, these data indicate that anti-CD40 transiently sensitized the liver to hepatoxicity from chemotherapy.

### An agonist CD40 antibody provokes immune activation in the liver.

**Figure 2:**
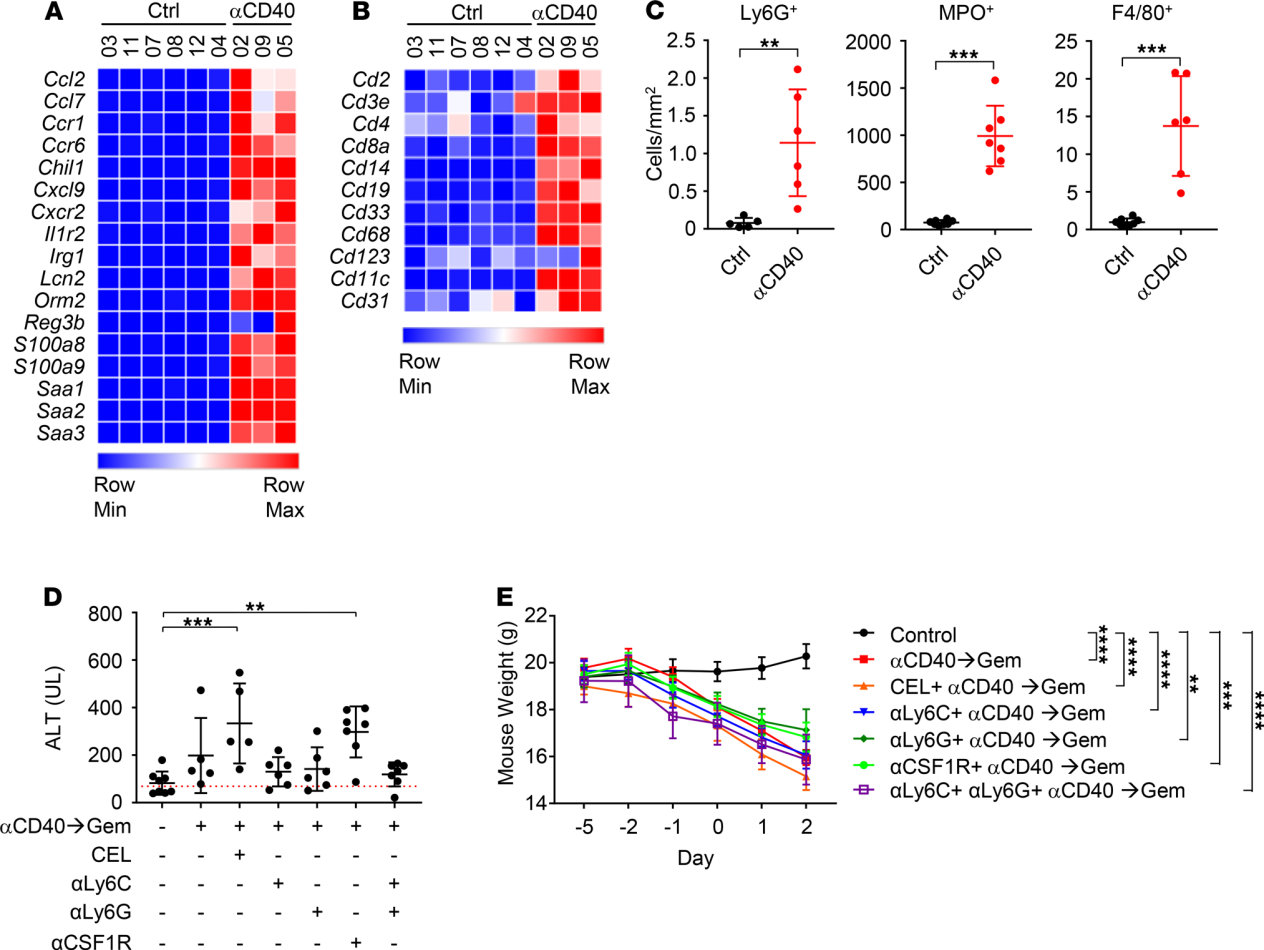
Myeloid cells are dispensable for hepatotoxicity triggered by chemoimmunotherapy. For **A**–**C**, mice (*n* = 3–6) were treated with control or αCD40, and liver was collected 2 days later for analysis. Shown are heatmaps from QuantSeq 3′ mRNA sequencing for (**A**) chemotaxis/acute inflammatory response-associated genes and (**B**) cell-specific markers. Heatmaps were generated from normalized FPKM values. (**C**) Quantification of cells expressing Ly6G and MPO, and clusters of cells (>900 mm^2^) expressing F4/80 in liver tissue detected by IHC. Mann-Whitney *U* tests were performed. *n* *=* 1 experimental replicate. For **D** and **E**, mice (*n* = 8 per group) were treated with αCD40 on day –2 and Gem on day 0. Analysis was performed on day 2. Myeloid-depleting agents were given as follows: CEL (days –4, –1); αLy6C (days –3, –2, –1, 0); αLy6G (days –3, –2, –1, 0); and αCSF1R (days –4, –2, 0). (**D**) ALT serum levels detected on day 2 after gemcitabine. Significance was tested with Kruskal-Wallis with Dunn’s multiple-comparison test. Significant comparisons with control are shown. *n* = 2 experimental replicates for control, αCD40→Gem, and αCD40→Gem+αCSF1R. *n* = 1 experimental replicate for all other groups. Red line indicates the upper range of the 95% CI for normal serum level of ALT derived from all experiments in the manuscript. (**E**) Mouse weight over time. Significance was tested with ordinary 1-way ANOVA with Dunnett’s multiple-comparison for weight on day 2. Data shown are mean ± SEM. *n* = 4 experimental replicates for control, αCD40→Gem, and αCD40**→**Gem+αCSFR. *n* = 2 experimental replicates for αCD40→Gem+CEL and αCD40→Gem+CEL+αLy6C. *n* = 1 experimental replicate for αCD40→Gem+αLy6G. All other data shown are mean ± SD. Gem, gemcitabine; αCD40, clone FGK45; CEL, clodronate-encapsulated liposomes; ALT, alanine aminotransferase; FPKM, fragments per kilobase of transcript per million mapped reads. **P < 0.01; ***P < 0.001; ****P < 0.0001.

To understand the impact of anti-CD40 on the liver, we next performed mRNA sequencing on RNA isolated from the livers of control and anti-CD40–treated mice 2 days after treatment. We identified 5464 differentially expressed genes (Supplemental Figure 3A) and found that genes upregulated in the liver after anti-CD40 were associated with response to IL-1, acute phase response, and chemokine signaling. Notably, genes encoding myeloid chemoattractants, including *Ccl2*, *S100a8*, *S100a9*, *Saa1*, *Saa2*, and *Saa3*, were upregulated in the livers of mice treated with anti-CD40 (Supplemental Figure 3B and Figure 2A). Consistent with increased expression of myeloid chemoattractants, we also found enrichment of myeloid cell markers (Figure 2B). We validated our results by IHC, which showed an increase in the number of granulocytes (Ly6G^+^), neutrophils (MPO^+^), and macrophage clusters (large F4/80^+^ areas) in the livers from mice treated with anti-CD40 (Figure 2C and Supplemental Figure 3C).

### Myeloid cells and MMPs are dispensable for hepatotoxicity triggered by chemoimmunotherapy.

We next investigated a role for myeloid cells in mediating hepatotoxicity triggered by anti-CD40. Prior work in tumor-bearing mice showed that the depletion of macrophages using anti-CSF1R antibodies abrogates hepatotoxicity observed when gemcitabine is administered 2 days after a CD40 agonist (21). Consistent with this, we found in tumor-bearing mice that anti-CSF1R diminished the number of necrotic lesions detected in the liver after chemoimmunotherapy (Supplemental Figure 4, A–C). However, in non–tumor-bearing mice, anti-CSF1R treatment did not significantly reduce necrotic lesions produced by chemoimmunotherapy (Supplemental Figure 4, B and C). Moreover, anti-CSF1R failed to prevent lethal weight loss or transaminitis in either tumor-bearing or non–tumor-bearing mice (Supplemental Figure 4, D and E). Based on these findings, we considered the possibility that other myeloid cell subsets might mediate hepatotoxicity induced by CD40 chemoimmunotherapy. To this end, we first tested the impact of administering clodronate-encapsulated liposomes (CELs), which broadly deplete liver-resident F4/80^+^ myeloid cells (Supplemental Figure 4, F and G). However, we found no significant effect of CEL on serum transaminitis, or weight loss induced by CD40 chemoimmunotherapy (Figure 2, D and E; and Supplemental Figure 4, H and I). We next tested the impact of depleting Ly6C^+^ and Ly6G^+^ myeloid cells using depleting antibodies. Similarly, we found that the depletion of these myeloid cell subsets did not prevent lethal weight loss triggered by chemoimmunotherapy (Figure 2E and Supplemental Figure 4I). In contrast, we did detect a trend toward reduced serum transaminitis, although a notable variability in transaminase levels was observed (Figure 2D and Supplemental Figure 4H). Based on this variability, we examined the relationship between weight loss and transaminase levels in treated mice and identified a significant correlation (Supplemental Figure 4, J and K). Thus, these findings show that the elimination of select myeloid cell subsets in mice was unable to prevent toxicity induced by CD40 chemoimmunotherapy.

We previously showed a role for MMPs, specifically MMP13, in the capacity of an agonist CD40 antibody to sensitize tumors to chemotherapy (15). Based on this biology, we considered whether altered MMP expression in the liver induced by anti-CD40 might sensitize the liver to toxicity in the setting of chemotherapy. We found that MMPs and tissue inhibitors of metalloproteinases were significantly altered in the liver 1, 2, and 5 days after anti-CD40 (Supplemental Figure 5, A and B). However, neither a broad-spectrum MMP inhibitor nor a selective inhibitor of MMP13 (15) was able to prevent the lethal weight loss triggered by chemoimmunotherapy (Supplemental Figure 5, C and D). Together, these data indicate that myeloid cells and MMPs were not the primary mediators of hepatotoxicity produced when chemotherapy was delivered 2 days after anti-CD40.

### IFN-γ and TNF produced in the setting of systemic CD40 activation sensitize the liver to hepatotoxicity.

**Figure 3:**
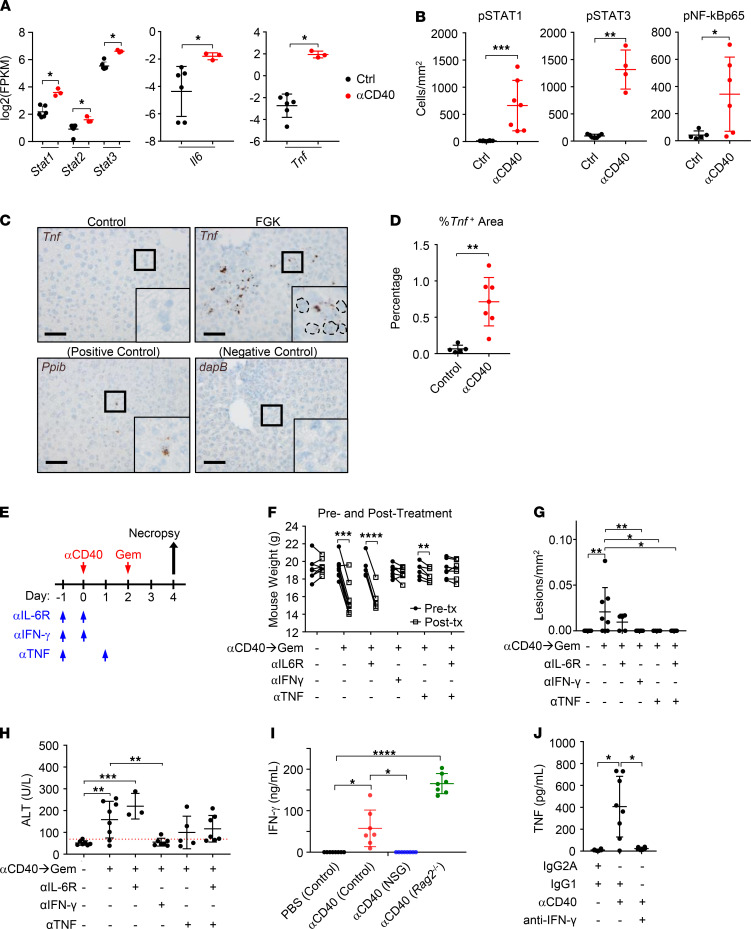
TNF is necessary for hepatotoxicity produced with chemoimmunotherapy. (**A**) RNA was extracted from bulk liver tissue of control- (Ctrl) or αCD40-treated mice 2 days after treatment. Gene expression for *Stat1*, *Stat2*, *Stat3*, *Il6*, and *Tnf* displayed as FPKM detected using QuantSeq 3′ mRNA sequencing. *n* = 3–6 mice/group, 1 experimental replicate. (**B**) Quantification by IHC of phosphorylated (p-) STAT1, p-STAT3, and p–NF-κBp65 protein expression in liver tissues collected 2 days after treatment with αCD40 compared with control. (**C**) Representative images and (**D**) quantification of *Tnf* expression detected by RNA-ISH in the liver 2 days after αCD40 treatment compared with control. Positive and negative controls for RNA-ISH are shown. Scale bars: 50 μm. Insets wre generated by zooming in on indicated 50 x 50 μm regions. (**A**–**D**) *n* = 8 mice/group, 1 experimental replicate. Mann-Whitney *U* tests were performed. (**E**) Study schema for **F–H**. *n* = 8 mice/group, 2 experimental replicates. (**F**) Mouse weight pretreatment and posttreatment on day 2. Paired 2-tailed *t* tests were performed. (**G**) Number of lesions/mm^2^ in the liver detected by H&E stain. (**H**) ALT serum levels on day 4. Red line indicates upper range of 95% CI for normal serum level of ALT derived from all experiments in the manuscript. (**I**) IFN-γ serum levels detected 24 hours after treatment with αCD40 in control, NSG, and *Rag2*^–/–^ mice. (**J**) TNF serum levels detected 1 day after treatment with αCD40 (compared with control). Anti–IFN-γ and isotype control (IgG1) were given on days –1 and 0. αCD40 and isotype control (IgG2a) were given on day 0. (**I** and **J**) *n* *=* 6–8 mice per group, 2 experimental replicates. Significance was tested using (**G** and **I**) Kruskal-Wallis with Dunn’s multiple-comparison test and (**H** and **J**) ordinary 1-way ANOVA with Dunnett’s multiple-comparison test. In **G**–**J**, comparisons with control and αCD40→Gem (**G** and **H**) or αCD40 (**I** and **J**) are shown. Data shown are mean ± SD. Gem, gemcitabine; αCD40, clone FGK45; ALT, alanine aminotransferase; FPKM, fragments per kilobase of transcript per million mapped reads. *P < 0.05; **P < 0.01; ***P < 0.001; ****P < 0.0001.

Anti-CD40 triggers CRS, which is characterized by an increase in the levels of multiple cytokines detected in the blood (14–16, 19). In prior studies, we showed that IL-6 levels (16) and IFN-γ levels (15) in the blood increase in patients (described in Figure 1) after treatment with a CD40 agonist. Further, we found in this same cohort of patients that anti-CD40 treatment induces a rapid but transient increase in TNF levels detected in the blood (Supplemental Figure 6A). Similar results have been reported for TNF in patients treated with a CD40 agonist as monotherapy (19). Based on these findings, we considered that signaling pathways activated by cytokines released in the setting of anti-CD40 might be responsible for hepatotoxicity. Gene set enrichment analysis of differentially expressed genes detected in the livers of mice 2 days after anti-CD40 showed enrichment for multiple immune-related pathways including IFN-γ response, IL-6/JAK/STAT3 signaling, and TNF signaling via NF-κB (Supplemental Figure 6, B–D). Consistent with this, we detected an increased expression of multiple genes associated with cytokine signaling including *Stat1*, *Stat2*, *Stat3*, *Il-6*, and *Tnf* (Figure 3A). We validated these findings by IHC, which showed an increased expression of phosphorylated STAT1 (p-STAT1), p-STAT3, and p–NF-κBp65 in the liver (Figure 3B and Supplemental Figure 6E). By RNA in situ hybridization (RNA-ISH), we also found an increased expression of *Tnf* in the liver after anti-CD40 treatment (Figure 3, C and D). Based on these findings, we next examined the impact of blocking the signaling of IFN-γ, TNF, and IL-6 on hepatotoxicity induced by treatment with chemotherapy administered 2 days after anti-CD40 (Figure 3E). We found that antibody neutralization of IFN-γ or TNF, but not IL-6, completely prevented the lethal weight loss and hepatic necrotic lesions triggered by chemoimmunotherapy (Figure 3, F and G; and Supplemental Figure 6, F and G). Further, serum transaminitis induced by chemoimmunotherapy was abrogated by anti–IFN-γ and reduced in mice receiving anti-TNF (Figure 3H and Supplemental Figure 6H). We tested a role for macrophages and other myeloid cell subsets as a source of TNF but found that the depletion of myeloid cells using anti-Ly6G and anti–Gr-1 antibodies did not alter the capacity of anti-CD40 to induce an increase in TNF levels detected in the blood (Supplemental Figure 6I). We also examined for cellular determinants of IFN-γ. Here, systemic release of IFN-γ after anti-CD40 treatment remained intact in *Rag2^–/–^* mice, which lack B cells and T cells, but was ablated in NOD-*scid* IL2Rγ^null^ (NSG) mice (Figure 3I). This finding implicates NK cells in the production of IFN-γ. Finally, neutralization of IFN-γ blocked the release of TNF detected in the serum after anti-CD40 (Figure 3J), demonstrating that TNF was a mediator downstream of IFN-γ induced by anti-CD40.

### TNF blockade prevents hepatotoxicity triggered by chemoimmunotherapy without impairing antitumor efficacy.

**Figure 4:**
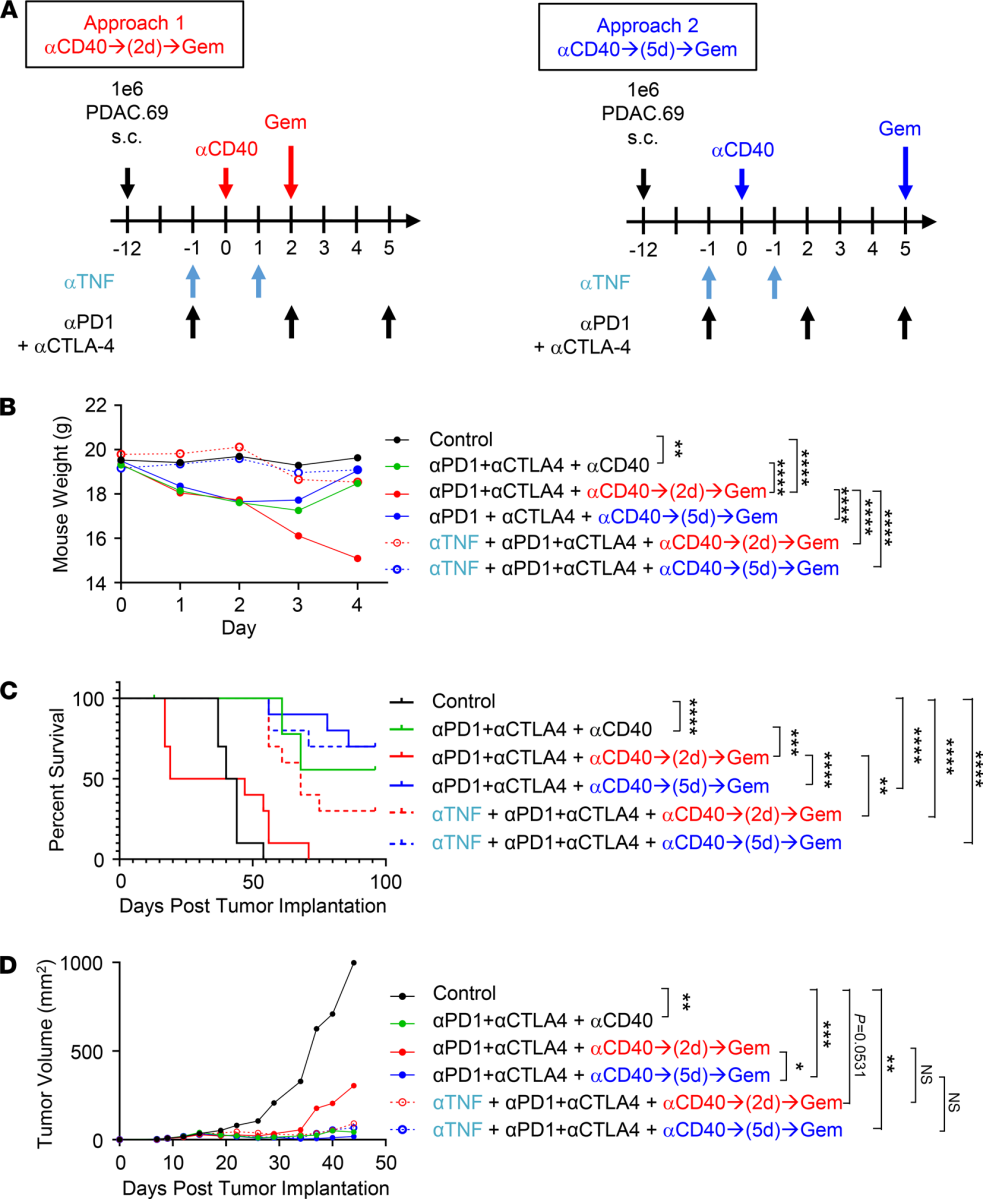
TNF blockade inhibits hepatotoxicity due to chemoimmunotherapy without affecting treatment efficacy. (**A**) Study schema used in **B**–**D**. Shown are (**B**) mean mouse weights over time, (**C**) overall survival, and (**D**) mean tumor growth curves. Statistical significance was determined using the following tests: in **B**, ordinary 1-way ANOVA with Dunnett’s multiple-comparison test was performed on weights on day 2; in **C**, Mantel-Cox test was used; and in **D**, Kruskal-Wallis with Dunn’s multiple-comparison test was performed on tumor volume on day 44. *n* *=* 10 mice/group. Data are representative of *n* = 2 experimental replicates. *P < 0.05; **P < 0.01; ***P < 0.001; ****P < 0.0001.

Because IFN-γ is essential for antitumor efficacy induced by an agonist CD40 antibody (13, 15), we next tested whether blockade of TNF might prevent hepatotoxicity without impairing antitumor efficacy. To do this, we investigated 2 therapeutic strategies (Figure 4A), wherein gemcitabine is administered either 2 or 5 days after anti-CD40 and in combination with immune checkpoint blockade including anti–CTLA-4 and anti–programmed cell death protein 1 (anti–PD-1) antibodies. As expected, we found that the delivery of gemcitabine chemotherapy 2 days after anti-CD40 produced lethal weight loss (Figure 4B). Notably, weight loss was abrogated by administering an anti-TNF neutralizing antibody. We also found in the absence of chemotherapy that anti-CD40 in combination with immune checkpoint blockade produced a significant but transient weight loss. This finding was not accentuated by incorporating gemcitabine 5 days after anti-CD40 but was prevented by incorporating an anti-TNF neutralizing antibody (Figure 4B).

We next examined the impact of an anti-TNF neutralizing antibody on the therapeutic efficacy of anti-CD40 administered in combination with chemotherapy and immune checkpoint blockade (Figure 4, C and D). Consistent with the induction of lethal weight loss, we found that the delivery of gemcitabine 2 days after anti-CD40 shortened median overall survival compared with control-treated mice (Figure 4, C and D; and Supplemental Figure 7, A and B). In contrast, incorporating TNF blockade into this treatment regimen led to a significant improvement in survival with 30% of mice achieving a complete response with cure. Notably, we also found that anti-TNF treatment had no detrimental impact on antitumor activity produced with gemcitabine chemotherapy delivered 5 days after anti-CD40 in combination with immune checkpoint blockade. Inclusion of gemcitabine chemotherapy 5 days after anti-CD40 was also not found to impair antitumor efficacy achieved with anti-CD40 in combination with immune checkpoint blockade. In summary, our data identify TNF as a therapeutic target for improving the safety of anti-CD40 treatment while preserving antitumor activity.

## Discussion

Immune-related toxicities remain a significant clinical challenge in the application of immunotherapy for the treatment of cancer (25, 26). For agonist CD40 antibodies, which have been in early-phase clinical trials for more than a decade, immune-related toxicities including CRS and hepatotoxicity have hampered clinical development. Our study demonstrates that hepatotoxicity triggered by an agonist CD40 antibody was mechanistically coupled to CRS. We show that CD40 activation sensitized the liver to chemotherapy-induced hepatotoxicity, resulting in transaminitis, necrotic liver lesions, and weight loss. We identified the upregulation of TNF signaling in the liver after treatment with a CD40 agonist and determined that TNF blockade was sufficient to abrogate toxicity without impairing antitumor efficacy in mice. Our findings provide a biological rationale to clinically explore TNF blockade as a preventative treatment approach for mitigating toxicity induced with anti-CD40 in patients.

As monotherapy, anti-CD40 shows minimal clinical activity in patients with cancer, which is the impetus for ongoing studies testing combinations with chemotherapy, radiation, and other immunotherapies. In this regard, promising activity has been reported with anti-CD40 in combination with chemotherapy for patients with metastatic PDAC (14, 16, 20, 27). However, immune-related toxicities, including CRS and hepatotoxicity, can be significant with agonist CD40 antibodies when given with chemotherapy (20). To this end, we and others have previously shown that the timing of administering chemotherapy with respect to anti-CD40 treatment can have implications on both treatment efficacy and toxicity (12, 15, 21). Specifically, chemotherapy when given prior to anti-CD40 is thought to elicit an immunogenic form of tumor cell death and the release of tumor antigens for presentation by APCs, which, when subsequently activated via CD40, prime tumor-specific T cells (2). In our studies conducted with mice, we found that administering chemotherapy prior to anti-CD40 did not worsen hepatotoxicity compared with anti-CD40 as monotherapy. This is consistent with a prior clinical study involving patients with advanced solid cancers, where anti-CD40 was shown to be well tolerated when given 2 or 7 days after chemotherapy (24). However, anti-CD40 can also sensitize tumors to chemotherapy by activating a myeloid-dependent immune response that resolves stromal elements of the tumor microenvironment involved in chemoresistance (15). In this regard, the timing of chemotherapy delivery after anti-CD40 treatment is critical and, if administered within 2 or 3 days of anti-CD40, can be lethal (15, 21). Practically, both strategies of sequencing chemotherapy with anti-CD40 are applicable to ongoing clinical trials given that standard-of-care chemotherapy is administered both before and after anti-CD40.

In our studies, we focused on defining the mechanism underlying toxicity induced by anti-CD40 when delivered prior to chemotherapy. We found that anti-CD40 sensitized the liver to toxicity from both gemcitabine and nab-paclitaxel chemotherapy, suggesting that the liver may be generally at increased risk for drug toxicity after systemic CD40 activation. In patients treated with gemcitabine in combination with anti-CD40, we observed grade 1–2 AST and ALT elevations as early as 2 days after anti-CD40 treatment, which persisted for nearly 2 weeks before returning to baseline levels. A recent phase Ib study combining a CD40 agonist with gemcitabine and nab-paclitaxel also reported transaminitis (20). However, in this study, grade 3 AST and ALT toxicities were observed in 6 of 15 patients (40%) treated at the recommended phase II dose for anti-CD40 (28, 29). Notably, grade 3 transaminitis is not common with gemcitabine in combination with nab-paclitaxel (28, 29), which implicates anti-CD40 as the major contributor to observed hepatotoxicity.

Our findings provide insight into mechanisms underlying CD40 immune-related toxicities. In prior studies, myeloid cells have been implicated as key mediators of liver toxicity induced by a CD40 agonist. Specifically, CD11b^+^Gr1^+^ myeloid cells and reactive oxygen species are identified as mediators of transaminitis induced by anti-CD40 as monotherapy in tumor-bearing mice (30). However, this report, which investigated the direct hepatotoxic effects of a CD40 agonist, differs markedly from our studies, which focused on mechanisms by which a CD40 agonist sensitizes the liver to subsequent toxicity from chemotherapy. In this regard, we found that CEL, which broadly deplete liver resident myeloid cells, were not able to prevent hepatotoxicity induced by anti-CD40 in combination with chemotherapy. We also studied the depletion of Ly6G^+^ and Ly6C^+^ cells using depleting antibodies and found that transaminitis tended to decrease, although there was no impact on weight loss. It remains possible that functional redundancy within the myeloid compartment might explain the inability of myeloid cell–depleting strategies to prevent toxicity induced by CD40 chemoimmunotherapy. However, this also illustrates the daunting challenge with intervening on cellular determinants as a strategy to ameliorate immune-related toxicities. To this end, CSF1R^+^ macrophages were previously shown to be required for the development of hepatic lesions marked by fibrin thrombi that form in tumor-bearing mice in response to anti-CD40 in combination with gemcitabine chemotherapy (21). Consistent with this, we also identified a role for CSF1R^+^ cells in the formation of hepatic lesions induced by anti-CD40 in combination with chemotherapy. Interestingly, this biology was observed only in tumor-bearing mice and not tumor-free mice. We have previously shown that cancer development can trigger an accumulation of myeloid cells and extracellular matrix deposition in the liver, which may explain this differential role for myeloid cells in mediating CD40-induced liver toxicity (31). However, even in tumor-bearing mice, anti-CSF1R treatment failed to mitigate treatment-induced weight loss or transaminitis triggered by CD40 chemoimmunotherapy. This finding is consistent with a recent phase Ib study that investigated anti-CSF1R (emactuzumab) in combination with a CD40 agonist (selicrelumab) and observed transaminitis in greater than 20% of patients, greater than 10% of which were grade 3 adverse events (32). Taken together, these findings support the need for alternative strategies, other than myeloid depletion, for dampening toxicities triggered by CD40 agonists. Moreover, combining myeloid-depleting with myeloid-activating strategies may be counterproductive for antitumor activity (33).

Systemic delivery of a CD40 agonist triggers a rapid cytokine release associated with bone marrow mobilization of myeloid cells and their trafficking into tumor tissues, where they subsequently facilitate the production of MMPs (15). Based on this biology, we considered a role for MMPs in sensitizing the liver to chemotherapy-induced toxicity. Indeed, we found significant changes in MMPs in the liver in response to anti-CD40. However, inhibiting MMPs had no effect on weight loss triggered by anti-CD40 in combination with chemotherapy. It is important to note that our studies assessing a role for MMPs in mediating anti-CD40 toxicity were conducted in tumor-free mice. Thus, like the case for CSF1R^+^ macrophages, it is possible that MMPs may contribute to toxicity in the context of a tumor-bearing host. However, our focus was to identify the main cellular and molecular determinants of toxicity independent of the context of a tumor. Notably, CD40 agonists have been used in combination with chemotherapy in the adjuvant setting (NCT02588443) for patients without detectable disease. Thus, we believe that our results are applicable to the use of CD40 agonists in the treatment of patients with active and measurable disease as well as patients with undetectable disease but who are at high risk for tumor recurrence.

Recent studies show a role for cytokines in mediating toxicities triggered by immunotherapy. For instance, IL-1 and IL-6 are associated with CRS and neurotoxicity triggered by CAR T cells (34, 35). Similarly, CRS induced by CD3 bispecific antibodies is initiated by T cell–dependent release of TNF, which subsequently triggers cytokine production, including IL-1 and IL-6, by monocytes and macrophages (36). TNF also mediates colitis caused by immune checkpoint blockade with anti–CTLA-4 and anti–PD-1 antibodies (37–39). Importantly, cytokine blockade, including anti-TNF and anti–IL-6 antibodies, has been found to ameliorate toxicities without impairing efficacy and is used for toxicity management in patients treated with immunotherapy (40). Based on these findings, we investigated a link between CRS and hepatotoxicity induced by anti-CD40 treatment. Our findings show that systemic CD40 activation triggers remarkable changes in the liver transcriptome with differential expression of 5464 genes. Notably, this remodeling of the liver is accompanied by an influx of myeloid cells and the activation of immune signaling pathways. Gene enrichment analyses suggested a key role for cytokines, particularly TNF, IL-6, and IFN-γ. We found that TNF and IFN-γ were required for hepatotoxicity induced by anti-CD40 in combination with chemotherapy. However, in contrast to CAR T cell therapy and CD3-bispecific antibodies, we found that CRS induced by anti-CD40 did not require T cells and IL-6 was dispensable for toxicity. To this end, our data implicate NK cells as the initial mediators of IFN-γ produced in response to systemic CD40 activation. Notably, we did not observe an increase in *Ifng* transcripts in the liver, suggesting that CD40 induction of IFN-γ is extrahepatic. In contrast, in situ hybridization studies showed that *Tnf* is upregulated in the liver in response to anti-CD40. We considered a role for myeloid cells as the cellular source of TNF but found that the depletion of myeloid cell subsets did not impact serum TNF levels. Thus, it is possible that nonmyeloid cell populations may contribute to TNF release. Nonetheless, we found that neutralizing IFN-γ antibodies blocked the increase in *Tnf* transcripts detected in the liver, which is consistent with the activation of IFN signaling pathways that we detected in the liver in response to anti-CD40. Together, our data support a model in which anti-CD40 induces extrahepatic release of IFN-γ, which then triggers the intrahepatic production of TNF and subsequent liver pathology. Further, our findings indicate that mechanisms underlying CRS may differ between myeloid-directed and T cell–directed immunotherapies but ultimately can converge on similar cytokine-dependent pathways that culminate in organ toxicity.

TNF can have opposing roles in cancer immunotherapy. Whereas TNF production locally within the tumor microenvironment can promote tumor killing, TNF has also been shown to induce apoptosis in mature T cells and limit T cell trafficking into tumors (41). TNF inhibitors are used in the clinical management of toxicities associated with the use of anti–CTLA-4 antibodies (25, 40). In mouse models, they have been shown to ameliorate age-associated hyperinflammatory cytokine responses produced by systemic immune activation of macrophages (42) as well as immune-related adverse events produced by anti-CD40 in the absence of chemotherapy (43). In our studies, we observed TNF-dependent hepatotoxicity triggered by CD40 chemoimmunotherapy even in young mice and independent of macrophages. Inhibition of TNF with neutralizing antibodies decreased toxicity. However, although generally well tolerated, anti-TNF medications have also been found to cause modest liver injury albeit at a relatively low incidence (44). Thus, clinical studies will be needed to further define the safety of anti-TNF medications when used in combination with anti-CD40 treatment. Nonetheless, we found that TNF blockade extended survival of mice, wherein chemotherapy was administered 2 days after a CD40 agonist with immune checkpoint blockade. By ameliorating toxicity, we were able to examine the relationship between the timing of chemotherapy after a CD40 agonist and antitumor efficacy. Notably, we found improved antitumor activity when chemotherapy was delayed to 5 days, compared with 2 days, after anti-CD40. This finding illustrates the importance of appropriate sequencing and timing of chemotherapy with immunotherapy.

Although our studies were performed using an implantable model of PDAC, we have previously reported activity with anti-CD40 in the treatment of tumors arising spontaneously in a genetic model of PDAC (12, 14). In addition, anti-CD40 has shown potential to combine with immune checkpoint blockade for the treatment of tumors arising in genetic mice (3). An ongoing study in patients with PDAC is evaluating anti-CD40 when combined with chemotherapy and immune checkpoint blockade as first-line therapy (20). Further, a retrospective analysis of survival outcomes for patients with PDAC treated with gemcitabine in combination with a CD40 agonist suggests benefit with the addition of anti-CD40 to chemotherapy (27). Our study now addresses the impact of delivering chemotherapy either before or after treatment with anti-CD40. Although we did not observe increased toxicity with administering chemotherapy prior to anti-CD40 in mice, this sequencing strategy may be detrimental for CD40-induced antitumor activity due to the capacity of chemotherapy to deplete monocytes and dendritic cells (27), the precise targets of anti-CD40. To circumvent this issue, we studied a role for chemotherapy delivered after a CD40 agonist. In this regard, we found that incorporating chemotherapy delivery 5 days after anti-CD40 did not hinder antitumor activity. However, we also found that inclusion of chemotherapy was not additive when used to treat PDAC tumors that are sensitive to immune checkpoint blockade. Consistent with this, a recent report showed the potential of anti-CD40 to combine with immune checkpoint blockade independent of cytotoxic therapy (45).

In summary, the results from this study show the capacity of prophylactic TNF blockade to prevent hepatotoxicity triggered by CD40 chemoimmunotherapy. We propose that a preventative approach is likely to be most beneficial, compared with the administration of anti-TNF upon clinical manifestation of immune-related toxicity, given the rapid release of TNF after systemic CD40 activation. Further, using a prophylactic strategy, we found that TNF blockade did not impinge on the therapeutic activity of anti-CD40 with or without chemotherapy and an improved toxicity was observed when anti-CD40 was combined with anti–PD-1 and anti–CTLA-4 antibodies even in the absence of chemotherapy. Thus, anti-TNF antibodies offer a clinically feasible approach for mitigating toxicity without impairing antitumor efficacy produced with agonist CD40 antibodies.

## Methods

### Clinical data.

Laboratory data were collected on patients with PDAC who were previously treated on a phase I clinical trial with gemcitabine on days 1, 8, and 15 and an agonistic CD40 antibody (CP-870,893) on day 3 of each 28-day cycle as previously reported (16). We analyzed clinical data for the patients enrolled in this study by extracting laboratory values from their electronic health records maintained by the Hospital of the University of Pennsylvania. Laboratory values that were extracted were AST and ALT serum levels corresponding to days 1, 2, 3, 4, 5, 8, and 15 of each 28-day cycle. Days with fewer than 3 patient data points were excluded from analysis.

### Cell lines.

PDA.7940B and PDA.69 cell lines (PDAC cells) were used in subcutaneous tumor models. These cell lines were derived from PDAC tumors that arose spontaneously in KPC mice, as previously described (15). Cell lines were cultured in DMEM (Corning) supplemented with 10% FBS (VWR), 83 μg/mL gentamicin (Thermo Fisher Scientific), and 1% GlutaMAX (Thermo Fisher Scientific) at 37°C, 5% CO_2_. Only cell lines that had been passaged fewer than 15 times were used for experiments, and trypan blue staining was used to ensure that cells with greater than 85% viability were used for studies. All cell lines used in our studies tested negative for mycoplasma contamination at the Cell Center Services Facility at the University of Pennsylvania.

### Animal experiments.

C57BL/6J mice were obtained from The Jackson Laboratory or bred in-house. In some experiments, mice from laboratory breeding colonies expressing Cre under the control of the Pdx promoter were used. In general, mice were monitored 3 times per week for general health and euthanized early based on defined endpoint criteria, including tumor volume greater than or equal to 1000 mm^3^, ascites, lethargy, loss of greater than or equal to 20% body weight, or other signs of sickness or distress.

For all animal studies, mice of similar age and sex were block randomized in an unblinded fashion. Sex-matched mice aged between 8 weeks and 12 weeks were used unless otherwise indicated. Sample sizes were estimated based on pilot experiments and were selected to provide sufficient numbers of mice in each group for statistical analysis.

For the administration of drugs, gemcitabine or nab-paclitaxel (120 mg/kg in 200 μL saline) was given by i.p. injection. MMP inhibitors WAY-170523 (0.02 mg) and actinonin (0.2 mg) were administered i.p. 30 minutes before anti-CD40 on days indicated in Supplemental Figure 5, as previously described (15). Antibodies used in in vivo studies were dosed as described in Supplemental Table 1. Antibodies were administered by i.p. injection in 200 μL sterile PBS via a 30-gauge needle. Approximately 10% of mice treated with anti-CD40 were excluded from analysis because treatment failed to produce the prototypical CD40-induced systemic inflammatory response that is characterized by a decrease in CD19^+^ B cells in the peripheral blood detected at 24 hours after treatment (16).

### AST/ALT analysis.

For animal studies, blood was collected by cardiac puncture at time of necropsy. Whole blood was centrifuged at 13,000 rpm for 20 minutes. Serum was collected and stored at –80°C until analyzed for AST and ALT levels by the Clinical Pathology Lab at the Ryan Veterinary Hospital at the University of Pennsylvania. The normal range for AST and ALT was defined by determining the 95% CIs for serum levels detected in untreated non–tumor-bearing mice across all studies conducted for this manuscript. For AST, the normal range was 179–294 U/L. For ALT, the normal range was 53–69 U/L.

### Microscopic analysis.

For preparation of FFPE sections, dissected tissues were fixed in 10% formalin for 24 hours at room temperature, washed twice with PBS, and then stored in 70% ethanol solution at 4°C until embedded in paraffin and sectioned at 5 μm. Automated IHC and RNA in situ hybridization were performed on FFPE sections using a Ventana Discovery Ultra automated slide staining system (Roche). Reagents were obtained from Roche and ACDBio (Supplemental Tables 2–4) and used according to manufacturer’s protocol. For manual H&E staining, sections were incubated 2 times for 2 minutes in xylene, 2 times for 2 minutes in 100% ethanol, 1 time for 2 minutes in 95% ethanol, and 1 time for 2 minutes in 70% ethanol. Sections were then rinsed in tap water for 2 minutes, stained in hematoxylin for 3 minutes, rinsed in tap water for 5 minutes, and stained in eosin for 2 minutes. Finally, sections were washed 3 times in 95% ethanol.

Images of tissue sections were acquired using an Aperio CS2 scanner system (Leica) or on a BX43 upright microscope (Olympus). Whole-slide scanned images were digitally quantified with custom algorithms created using Visiopharm Software (Version 2019.07). For MPO, F4/80, p-STAT1, and p-STAT3 analyses, regions of interest (ROIs) were determined by a “tissue detect” algorithm to identify the liver, and positively stained cells were quantified within each ROI. Absolute cell counts (or cluster counts in the case of F4/80) were normalized to the ROI area and reported as density (cells per mm^2^). For Ly6G and p–NF-κBp65, the edges of the liver were excluded due to staining artifacts, and then the same procedure was followed. For RNA-ISH analysis, 10 original magnification 40× images per sample were analyzed, and the percentage area of positive staining was determined.

### Flow cytometry.

Peripheral blood (10 μL) was collected via tail vein bleed. Cells were resuspended in ACK lysing buffer (Life Technologies, Thermo Fisher Scientific) at room temperature for 5 minutes to remove red blood cells. After washing 3 times with PBS, cells were stained using Fixable Aqua Dead Cell Stain Kit (Life Technologies, Thermo Fisher Scientific) following the manufacturer’s protocol. For characterization of immune cell subsets, cells were washed with PBS containing 0.2 mM EDTA with 2% FBS and stained with appropriate antibodies (Supplemental Table 2). Cells were fixed with 3% formaldehyde in PBS. Last, cells were washed 3 times with PBS containing 0.2 mM EDTA with 2% FBS and examined using a FACSCanto II (BD Biosciences). Flow cytometric data were analyzed using FlowJo version 10.2.

### Cytometric bead array analysis for IFN-γ and TNF-α.

Mice were bled retro-orbitally 1 day after treatment, and serum was collected by centrifuge at 10,000*g* for 15 minutes at room temperature. Cytokine levels were measured by a cytometric bead array kit for mouse IFN-γ and TNF (BD Biosciences) using a FACSCanto II (BD Biosciences).

### RNA sequencing and analysis.

Mouse organs were stored in TRIzol (Thermo Fisher Scientific) at –80°C until analysis. Samples were thawed on ice and allowed to equilibrate to room temperature before RNA was isolated using the RNeasy Mini Kit (QIAGEN) following the manufacturer’s protocol. RNA was submitted to the Genomics Facility at the Wistar Institute. After the quality of RNA was assessed using a 2100 Bioanalyzer (Agilent), samples were prepared using a QuantSeq 3′ mRNA-Seq Library Prep Kit FWD for Illumina (Lexogen) following the manufacturer’s protocol and analyzed on a NextSeq 500 sequencing system (Illumina). FastQ files were uploaded to the BaseSpace Suite (Illumina) after sequencing and aligned to the *Mus musculus* 10 (mM 10) genome using the STAR aligner within the RNA-Seq Alignment (version 1.1.0) application. The maximum allowed mismatches were set to 14 bases as recommended by manufacturer. Output.bam/.bai files were analyzed by CuffDiff using the Cufflinks Assembly & DE (version 2.1.0) application in the BaseSpace Suite to determine differentially expressed genes (DEGs) between experimental groups. Significant DEGs (adjusted *P* <.05) were used to generate an expression heatmap in Morpheus (Broad Institute, https://software.broadinstitute.org/Morpheus).

DEGs were analyzed using the ClueGO (version 2.5.4) and Cluepedia (version 1.5.4) applications (46) within the Cytoscape software (version 3.7.1) (47). Functional grouping of resultant biological processes was performed according to the output κ score. Morpheus (https://software.broadinstitute.org/morpheus) was used to generate an expression heatmap of significant genes from ClueGo biological processes.

Gene set enrichment analysis (version 4.0.3) was performed using Gene Ontology data (downloaded October 18, 2019) (48, 49) to determine biological processes that were differentially enriched in experimental groups (50). Morpheus was used to generate an expression heatmap of the enriched gene sets.

### RNA isolation and quantitative reverse-transcription PCR.

For RNA studies, murine bulk liver tissue was harvested and lysed in TRIzol at 4°C. Samples were stored at –80°C until analysis. Samples were then thawed on ice and allowed to equilibrate to room temperature before RNA was isolated using the QIAGEN RNeasy Mini kit, according to manufacturer protocol. RNA was collected in RNase-free water and quantified using a NanoDrop spectrophotometer (Thermo Fisher Scientific). RNA was converted to cDNA for quantitative reverse-transcription PCR using the High-Capacity cDNA Reverse Transcription Kit according to manufacturer protocol (Applied Biosystems, Thermo Fisher Scientific).

Murine-specific *Mmp* and *Timp* primers for quantitative reverse-transcription PCR were designed using the Primer 3 online program (http://frodo-wi.mit.edu) and synthesized by Integrated DNA Technologies. Primer sequences are listed in Supplemental Table 5. Relative quantification of all products was measured using TaqMan assays (Applied Biosystems, Thermo Fisher Scientific). Expression was normalized to *Gapdh* and the relative gene expression for each gene was calculated using the ΔCT formula. The fold increase or decrease in expression for samples obtained from treated mice was calculated as a ratio over the expression observed in samples obtained from control mice (ΔΔCT).

### Data availability.

Sequencing data have been deposited in the National Center for Biotechnology Information’s Gene Expression Omnibus under access number GSE156160.

### Statistics.

Statistical significance was calculated using Prism (GraphPad Software, version 7) unless otherwise indicated. Paired group comparisons were evaluated using 2-tailed Wilcoxon’s matched pairs signed-rank test or 2-tailed paired *t* test. Unpaired comparison tests between 2 groups were performed using 2-tailed Mann-Whitney *U* test. For multiple-comparison testing, Gaussian distribution was evaluated using the Shapiro-Wilk test. One-way ANOVA with Dunnett’s multiple-comparison test or Brown-Forsythe and Welch’s 1-way ANOVA tests with Dunnett’s T3 multiple comparisons test were used for comparison between more than 2 groups with Gaussian distribution. Kruskal-Wallis test with Dunn’s multiple-comparison test was used for comparison among more than 2 groups without Gaussian distribution. Outliers were detected with the Grubbs outlier test and excluded from the analysis. Comparison of Kaplan-Meier overall survival curves was performed using log rank (Mantel-Cox) test. Unless otherwise stated, data shown are mean ± SD. *P* values less than 0.05 were treated as significant. The experiments were not randomized and the investigators were not blinded to allocation during experiments and outcome assessment, unless otherwise stated.

### Study approval.

For human studies, written informed consent was required, and the study was approved by the University of Pennsylvania Institutional Review Board. For animal studies, protocols were reviewed and approved by the IACUC of the University of Pennsylvania.

## Author contributions

MLS, GLB, JL, VMH, YL, KBL, JWL, THB, and AH contributed to study design, data analysis, and interpretation. MLS, KG, HC, JL, ET, VMH, YL, MIM, THB, and KBL performed experiments. MLS, JL, VMH, YL, MIM, and MG created figures. MLS and GLB wrote the manuscript. All authors reviewed and approved the final manuscript.

## Supplementary Material

Supplemental data

## Version 1 06/08/2021

In-Press Preview

## Version 2 07/22/2021

Electronic publication
