# Supplementary material for: TNF blockade uncouples toxicity from antitumor efficacy induced with CD40 chemoimmunotherapy
Source: JCI Insight. 2021 Jul 22;6(14):e146314. doi: 10.1172/jci.insight.146314 (PMC8410039; doi:10.1172/jci.insight.146314)
Supplement: Supplemental data [file jciinsight-6-146314-s120.pdf]

Application	Target antigen/cell	Target species	Host species	Clone	Conjugation	Dose	Vendor	Catalog #
In vivo	Rat IgG1 isotype control	N/A	Rat	HRPN	Unconj.	0.5mg	BioXCell	BE0088
	Rat IgG2A isotype control	N/A	Rat	2A3	Unconj	0.1mg	Bioxcell	BE0089
	CD40	Mouse	Rat	FGk45	Unconj	0.1mg	BioXCell	BE0016-2
	CSF1R	Mouse	Rat	AFS98	Unconj	1 mg for first dose, then 0.5mg	BioXCell	BE0213
	F4/80^{+} myeloid cells	N/A	N/A	N/A	Unconj	10 μL/g of weight	Liposoma	C-010
	Ly6C	Mouse	Rat	Monts-1	Unconj	0.5mg first dose, then 0.2mg	BioXCell	BE0203
	Ly6G	Mouse	Rat	1A8	Unconj	0.5mg first dose, then 0.2mg	BioXCell	BP0075-1
	IL-6R	Mouse	Rat	15A7	Unconj	0.2 mg	BioXCell	BE0047
	IFN-γ		Rat	H22	Unconj	0.5mg	BioXCell	BE0312
	TNF-α	Mouse	Rat	XT3.11	Unconj	0.2mg	BioXCell	BE0058
	Gr-1	Mouse	Rat	RB6-8C5	Unconj	0.5mg	BioXCell	BE0075
	PD-1	Mouse	Rat	RMP1-14	Unconj	0.2mg	BioXCell	BE0146
	CTLA-4	Mouse	Rat	XMG1.2	Unconj	0.2mg	BioXCell	BE0164

Supplementary Table 1 | Antibodies used in vivo. N/A, not applicable; Unconj, unconjugated.

Application	Target antigen/cell	Target species	Host species	Clone	Conjugation	Dilution	Vendor	Catalog #
FCM	CD45	Mouse	Rat	30-F11	FITC	1:100	BD Biosciences	553080
	CD19	Mouse	Rat	5D5	PE-Cy7	1:100	Biolegend	115520
	CD3e	Mouse	Hamster	145-2C11	PerCP	1:200	BD Biosciences	551163
	CD4	Mouse	Rat	RM4-5	APC	1:100	eBioscience	17-0042-82
	CD8b	Mouse	Rat	YTS156.7.7	FITC	1:100	Biolegend	126606
	CD44	Mouse	Rat	IM7	PE	1:100	eBioscience	12-0441-82
	CD62L	Mouse	Rat	MEL-14	APC-Cy7	1:100	Invitrogen	A15409
	PD1	Mouse	Rat	RMP1-30	PE-Cy7	1:100	Biolegend	109110
	Ki-67	Mouse	Rat	16A8	Pacific Blue	1:100	Biolegend	652422
	Dead Cells	Mouse	N/A	N/A	Aqua	1:600	Fisher	L34966
RNA ISH	*Tnf*	Mouse	N/A	N/A	Unconj	No dilution	ACDBio	311089
	Ppib	Mouse	N/A	N/A	Unconj	No dilution	ACDBio	313919
	dapB	Bacillus subtilis	N/A	N/A	Unconj	No dilution	ACDBio	312038

Supplementary Table 2 | Antibodies and probes used in flow cytometry and RNA ISH. FCM, flow cytometry; ISH, in situ hybridization; N/A, not applicable; Unconj, unconjugated.

Application	Target antigen		Target species	Host species	Clone	Conjugation	Dilution	Vendor	Catalog #
Automated IHC	Primary	Ly6G	Mouse	Rat	1A8	Unconj	1:100	BioXCell	BE0075-1
		MPO	Mouse	Rabbit	Polyclonal	Unconj	1:25	Abcam	Ab9535
		F4/80	Mouse	Rabbit	D2S9R	Unconj	1:250	Cell Signaling	70076S
		pSTAT1	Mouse	Rabbit	58D6	Unconj	1:100	Cell Signaling	9167S
		pSTAT3	Mouse	Rabbit	D3A7	Unconj	1:100	Cell Signaling	9145L
		pNF-kBp65	Mouse	Rabbit	Polyclonal	Unconj	1:100	Abcam	Ab194726
	Secondary	IgG	Rat	Goat	Polyclonal	HRP	No dilution	Ventana	760-4457
		IgG	Rabbit	Goat	Polyclonal	HQ	No dilution	Ventana	760-4815
		HQ	N/A	N/A	N/A	HRP	No dilution	Ventana	760-4820

Supplementary Table 3 | Antibodies used in automated IHC. HRP, horseradish peroxidase; HQ, OptiView HQ Universal Linker; IHC, immunohistochemistry; N/A, not applicable; Unconj, unconjugated.

Chromagen	Application used	Vendor	Catalog #
DAB (brown)	RNA ISH	Ventana	760-225
DAB (brown)	Automated IHC	Ventana	760-225

Supplementary Table 4 | Chromogens used in experiments. DAB, 3,3’-diaminobenzidine, IHC, immunohistochemistry; ISH, *in situ* hybridization.

Gene	Left Primer Sequence	Right Primer Sequence
Mmp2	ACCCAGATGTGGCCAACTAC	AAAGCATCATCCACGGTTTC
Mmp3	TGGAGATGCTCACTTTGACG	AGCCTTGGCTGAGTGGTAGA
Mmp7	CGGAGATGCTCACTTTGACA	ACCGGGAACAGAAGAGTGAC
Mmp8	CTTTCAACCAGGCCAAGGTA	GAGCAGCCACGAGAAATAGG
Mmp9	AGACGACATAGACGGCATCC	TGGGACACATAGTGGGAGGT
Mmp10	CAGTTGGAGaACACGGAGACT	TGTCCATTTCTCATCATCATCG
Mmp11	GACGCTGGGAGAAGACAGAC	GTGGGGTCACTTCACTCCATA
Mmp12	GCTGTCACAACAGTGGGAGA	ATACCAGATGGGATGCTTGG
Mmp13	TGGACCTTCTGGTCTTCTGG	TGGGCAGCAACAATAAACAA
Mmp14	CCAGTGGATGGACACAGAGA	AGAGGGCCGAGAGGTAGTTC
Mmp15	CAGGAAAGGCATGGAACAAT	ACCAATGGTGTGACCTGCTC
Timp1	GCATCTGGCATCCTCTTGTT	TGGGGAACCCATGAATTTAG
Timp2	AAGCAGTGAGCGAGAAGGAG	GGGGGCCGTGTAGATAAACT
Timp3	GTGGGAAAGAAGCTGGTGAA	AGAGGCTTCCGTGTGAATGT

Supplementary Table 5 | Primer sequences

Supplementary Figure 1

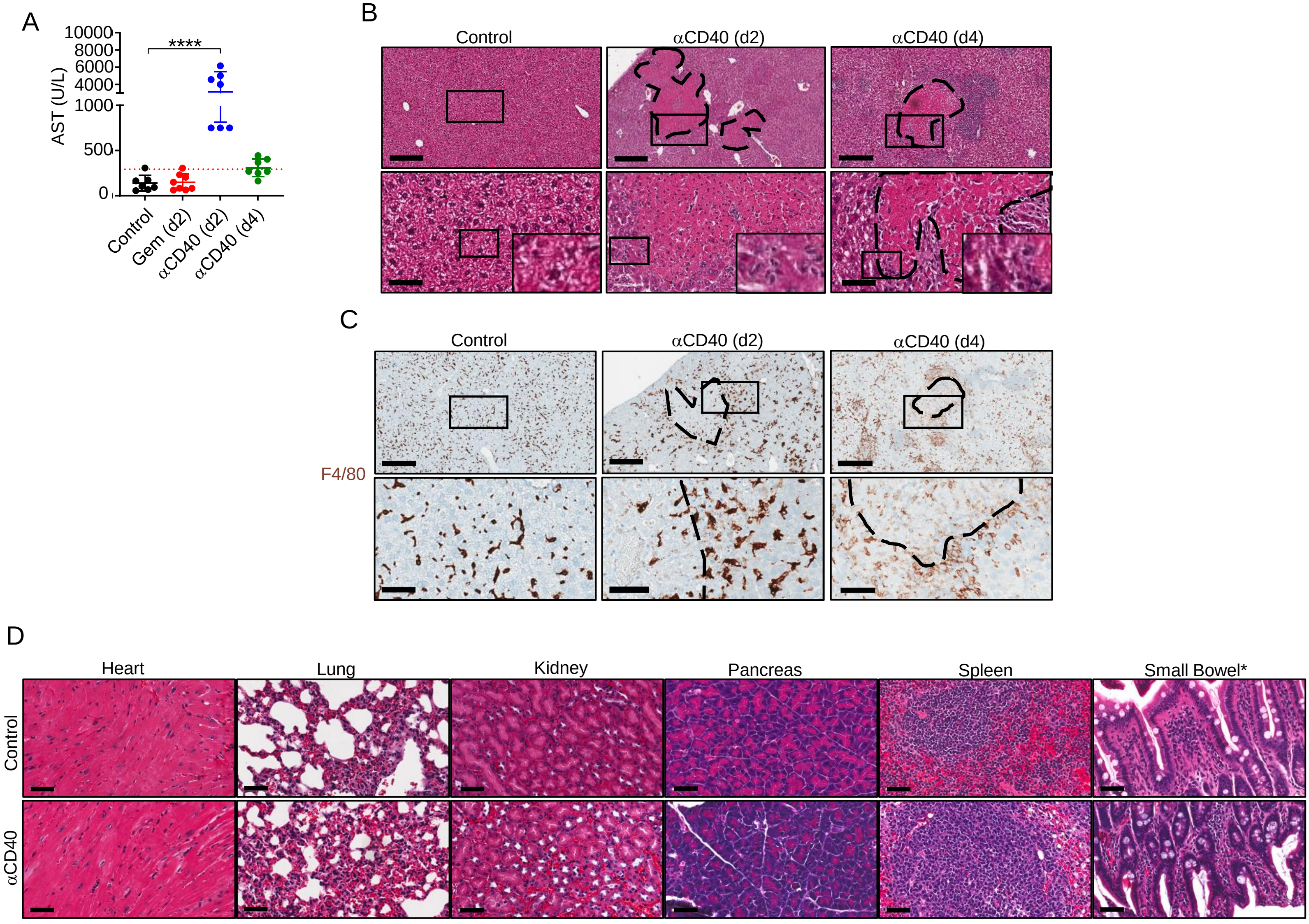

Supplementary Figure 1. Systemic CD40 activation induces hepatotoxicity. Mice were treated with gemcitabine (Gem) and αCD40 (FGK45) as in **Figure 1D**. (**A**) AST serum levels were detected on the day of analysis (shown in parentheses) Data shown are mean +/- SD. Significance was determined by ordinary one-way ANOVA with Dunnett's multiple comparisons test. Red line indicates upper range of 95% confidence interval for normal serum level of AST derived from all experiments in the manuscript. (**B-C**) Representative images of (**B**) H&E or (**C**) F4/80 stained tissues from mice treated as indicated. Day of analysis is shown in parentheses. Dashed lines indicate necrotic lesions in livers. In (**B**), scale bars = 200μm (top row) and 50μm (bottom row). In (**C**), scale bars = 300μM (top row) or 60μm (bottom row). (**D**) Representative images of H&E stained tissues collected at 2 days after control or αCD40 treatment. *, image of small bowel was collected at 1 day after αCD40. Scale bars = 50μm. Data are representative of ≥1 experimental replicates.

Supplementary Figure 2

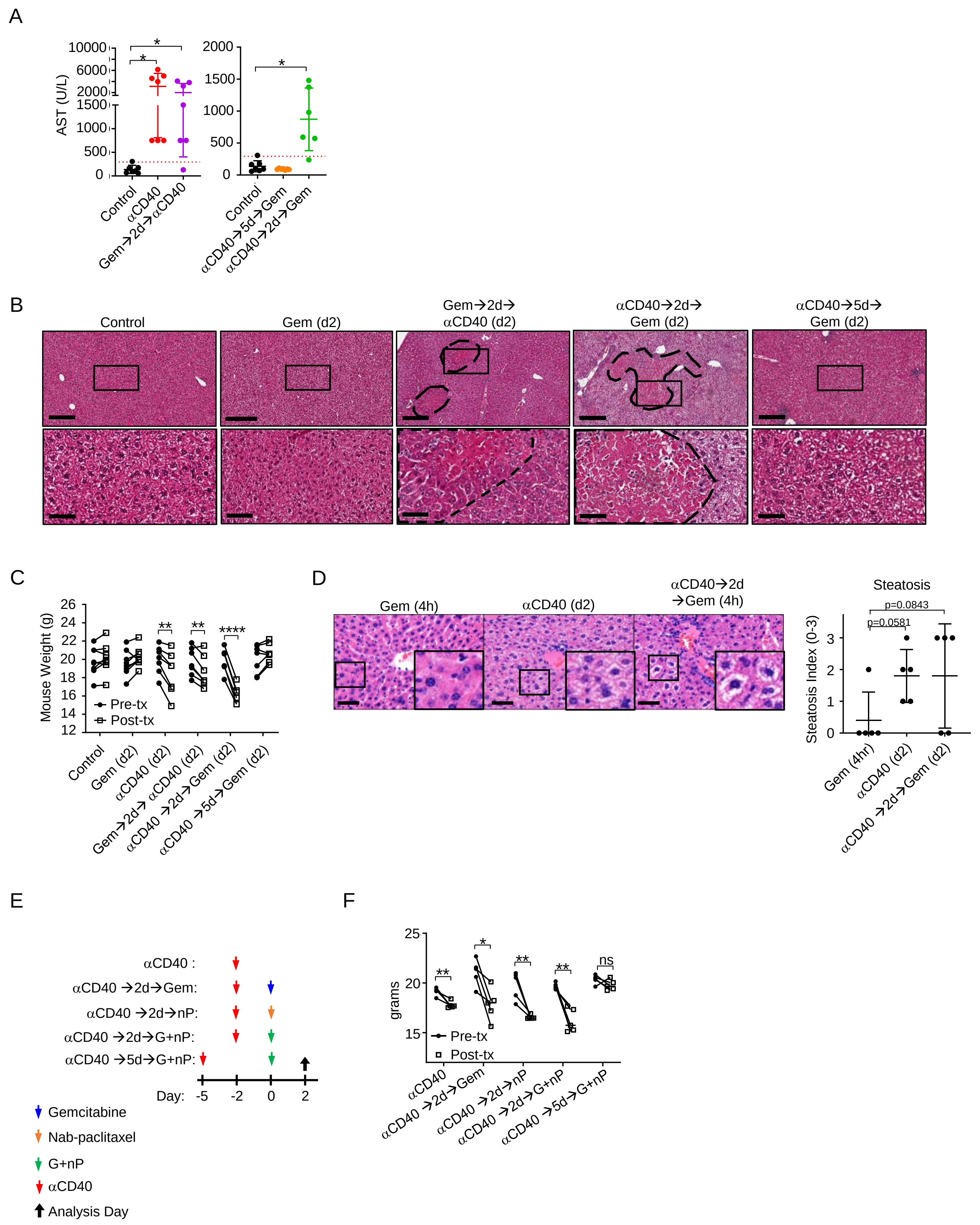

Supplementary Figure 2. The timing of chemotherapy sequencing with anti-CD40 impacts development of hepatotoxicity. Mice were treated with gemcitabine (Gem) and αCD40 (FGK45) as in **Figure 1H or 1L**. (**A**) AST serum levels. Data shown are mean +/- SD. In the *left panel*, serum AST is measured on day 2 after receiving αCD40; and in the *right panel*, serum AST is measured on day 2 after receiving gemcitabine. Red lines indicate upper range of 95% confidence interval for normal serum level of AST derived from all experiments in the manuscript. Significance was determined by Brown-Forsythe and Welch ANOVA tests with Dunnet's T3 multiple comparisons test (**B**) Representative images of H&E stained tissues from mice treated as indicated. Day of analysis is shown in parentheses. Dashed lines indicate necrotic lesions in livers. Scale bars = 200μm (top row) or 50μm (bottom row). (**C**) Mouse weight measured pre- and post-treatment at indicated day (shown in parentheses). Paired *t* tests were performed. (**D**) Mice were treated as indicated with analysis performed at time (shown in parentheses) after treatment. Hepatic steatosis was scored as 0, none; 1, minimal; 2, moderate, and 3, severe. Scale bars = 50μm. Kruskal-Wallis with Dunn's multiple comparisons test was performed. (**E**) C57Bl/6 mice (n=5 per group) were treated with FGK45 (αCD40), gemcitabine (Gem; G), or nab-paclitaxel (nP). (**F**) Mouse weight pre- and post- treatment. Paired *t* tests were performed. *, $p<0.05$, **, $p<0.01$.

Supplementary Figure 3

A

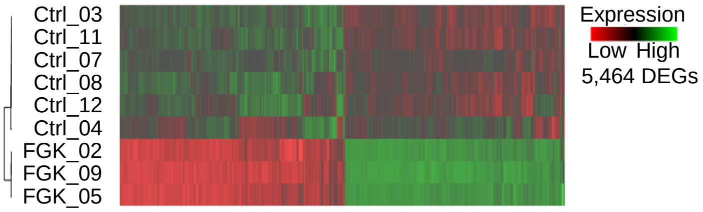

B

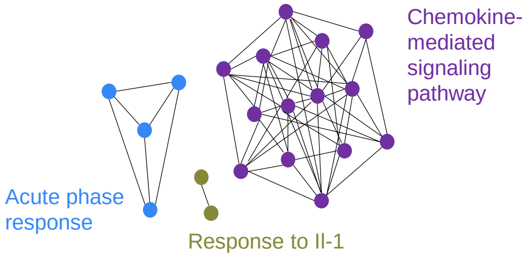

Gene Ontology Term	Term p-value (Bonferroni Corrected)
Acute-Phase Response	2.20E-06
Response to Stilbenoid	5.48E-06
Acute Inflammatory Response	5.56E-06
Chemoattractant Activity	4.86E-04
Response to Interleukin-1	3.35E-07
Cellular Response to Interleukin-1	1.42E-06
Chemokine-Mediated Signaling Pathway	6.33E-07
Leukocyte Migration Involved in Inflammatory Response	1.16E-06
Cellular Response to Chemokine	1.17E-06
Response to Chemokine	1.17E-06
Neutrophil Chemotaxis	2.00E-06
Neutrophil Migration	4.44E-06
Granulocyte Chemotaxis	5.56E-06
Positive Regulation of Leukocyte Chemotaxis	1.77E-05
Chemokine Receptor Activity	1.25E-04
G Protein-Coupled Chemoattractant Receptor Activity	1.25E-04
C-C Chemokine Receptor Activity	1.34E-04
Cytokine Receptor Activity	2.19E-04
Chemokine Activity	3.44E-04
Monocyte Chemotaxis	4.58E-04

C

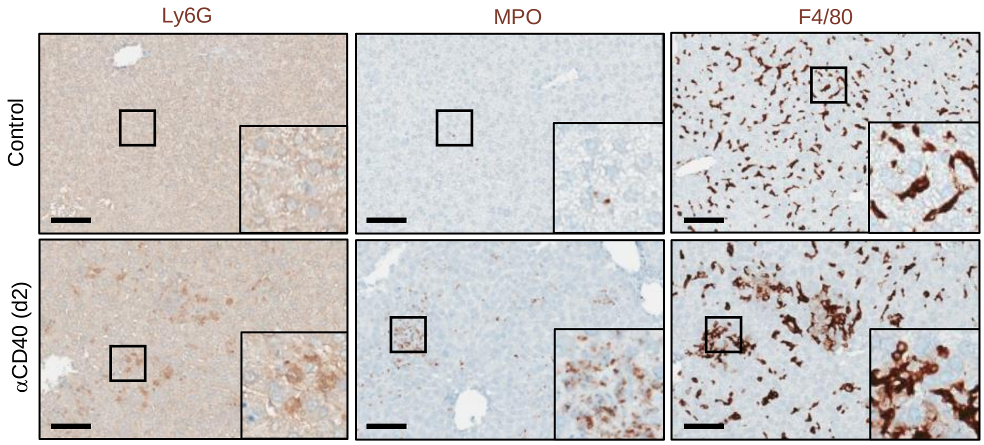

Supplementary Figure 3. Systemic CD40 activation upregulates immune signaling genes in the liver. (**A**) Heat map generated from normalized FPKM values obtained from QuantSeq 3′ mRNA sequencing of bulk liver tissue collected from wild-type mice at 48 hours after treatment with the CD40 agonist FGK45 (compared to control). Shown are 5,464 differentially expressed genes (DEGs) detected in αCD40-treated mice (FGK, *n* = 3) compared to control mice (Ctrl, *n* = 6). (**B**) Enriched biological processes for genes with >32-fold increased expression in livers from αCD40-treated mice. (**C**) Mice were treated as in Figure 2C. Shown are representative images of livers stained for F4/80, Ly6G, or MPO. Scale bars = 100μm. Data shown are 1 experimental replicate.

Supplementary Figure 4

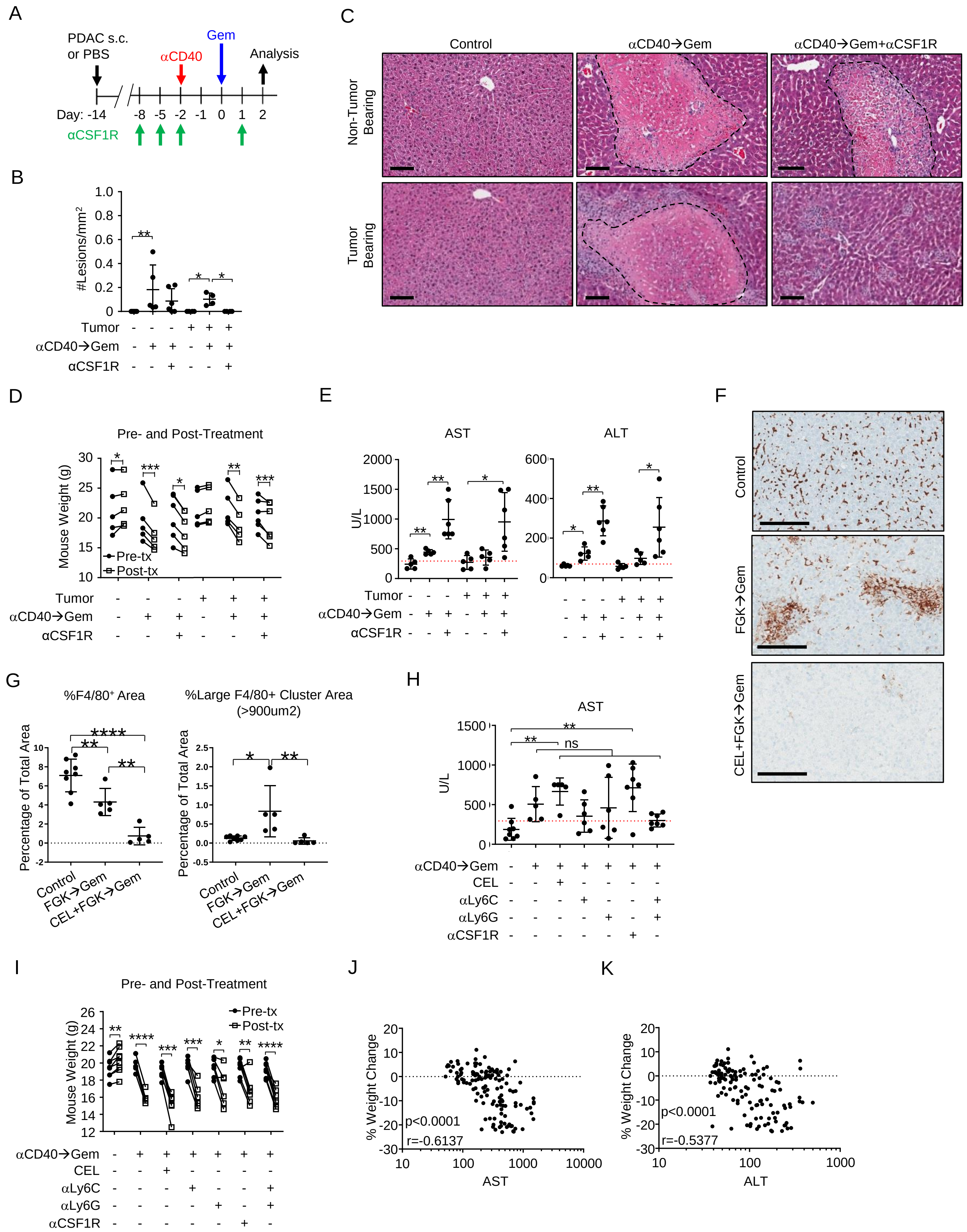

Supplementary Figure 4. Myeloid cells accumulate in the liver but are not required for chemoimmunotherapy-induced hepatotoxicity. (**A**) Study design for **B-E**. Mice were subcutaneously injected with 5e5 7940B cells (PDAC) or PBS treated as indicated. *n*=5-6 mice/group, 1 experimental replicate (tumor bearing) and 2-3 experimental replicates (non-tumor bearing). (**B**) Quantification and (**C**) Representative H&E images of necrotic lesions (indicated by dashed lines) in the liver. Scale bars=100μm. Kruskal-Wallis with Dunn's multiple comparisons tests were performed. (**D**) Mouse weight measured pre- and post-treatment on day 2. Paired t tests were performed. **(E)** AST (left) and ALT (right) serum levels detected at necropsy on day 2. Brown-Forsythe and Welch ANOVA tests with Dunnet's T3 multiple comparisons tests were performed. (**F-I)** Mice were treated as in Figure 2D. **(F)** Livers were formalin fixed and paraffin embedded and stained for F4/80 (brown). Scale bar=200μm. **(G)** Quantification of the area of F4/80$^+$ cells by Visiopharm software. Left: total F4/80$^+$ staining. Ordinary one-way ANOVA with Dunnett's multiple comparisons test was performed. Right: F4/80$^+$ clusters (>900μm^2). Kruskal-Wallis with Dunn's multiple comparisons test was performed. (**H**) AST serum levels 2 days after gemcitabine treatment. Kruskal-Wallis with Dunn's multiple comparisons test was performed. **G-H)** *n*=1 experimental replicate. **E, H)** red line indicates upper range of 95% confidence interval for normal serum level of AST derived from all experiments in the manuscript. (**I**) Mouse weight pre- and post-treatment on day 2. Paired *t* tests were performed. Experimental replicates: *n*=4 (control, αCD40→Gem, and αCD40→Gem+αCSFR); *n*=2 (αCD40→Gem+CEL and αCD40→Gem+αLy6C); *n*=1 (αCD40→Gem+αLy6G). (**J-K**) AST or ALT serum levels as a function of percent weight loss from all experimental groups treated with or without FGK45 at 4 days before necropsy. Some mice received gemcitabine, neutralizing antibodies, or myeloid depleting agents. Spearman correlation analysis was performed. p<0.0001 For **B, E, H,** comparisons to control and αCD40→Gem are shown. All error bars shown represent mean +/- SD.

Supplementary Figure 5

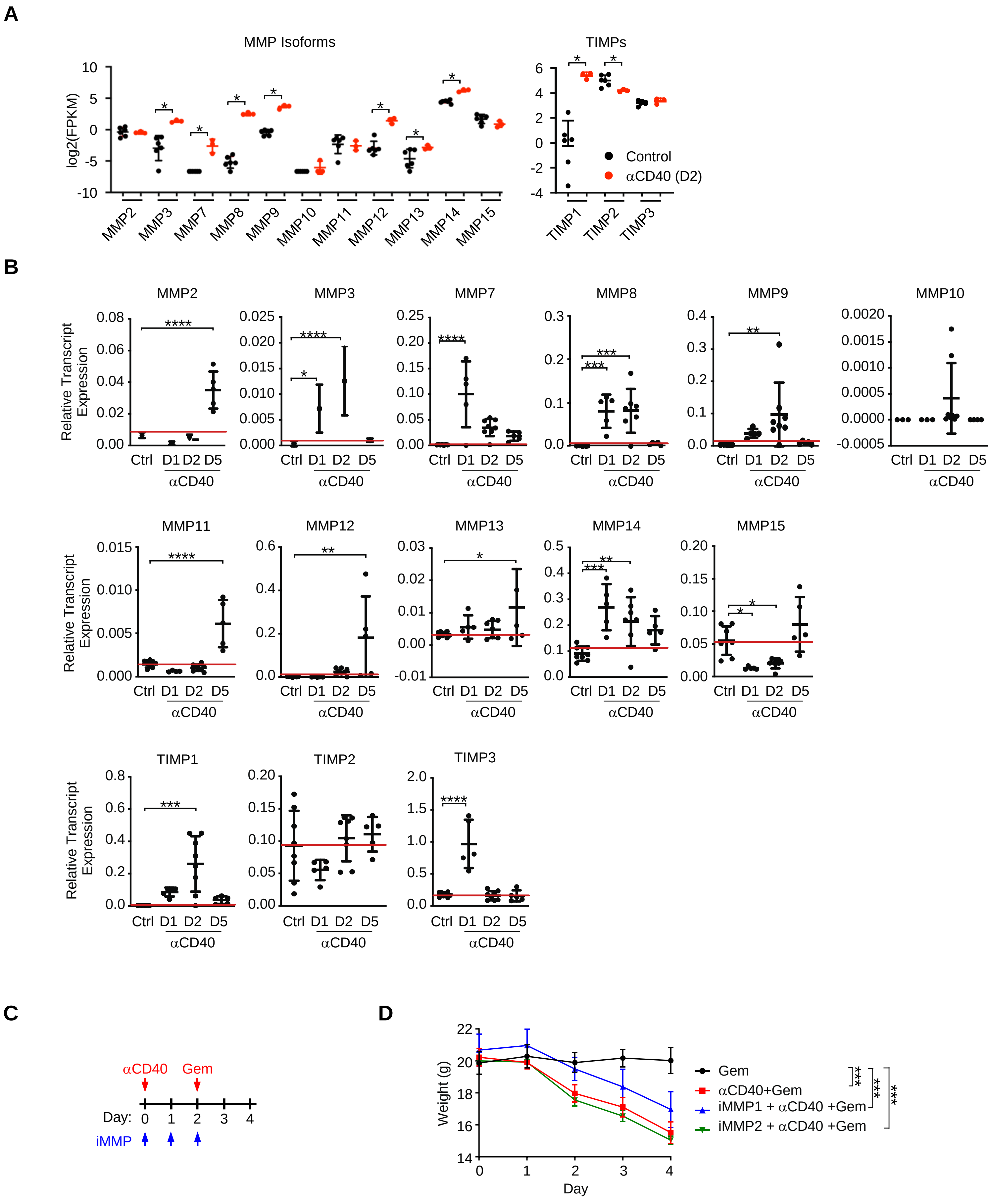

Supplementary Figure 5. Anti-CD40 induced changes in liver MMPs are dispensable for hepatoxicity. (**A**) RNA was extracted from bulk liver tissue collected from mice treated with control or FGK45 (αCD40) at 2 days post treatment. *n*=3-6 mice per group. Gene expression is shown as fragments per kilobase of transcript per million mapped reads (FPKM) detected using QuantSeq 3′ mRNA sequencing. Mann Whitney tests were performed. (**B**) Relative transcript expression of MMPs and TIMPs detected in livers of mice at 1, 2, and 5 days post treatment with αCD40 compared to control (Ctrl). *n*=10 mice per group. Ordinary one way ANOVA with Dunnett's multiple comparisons tests were performed. For **A-B**, data shown are mean+/-SD. (**C**) Study schema for **D**. Mice (n=5/group) were treated with αCD40 and gemcitabine (Gem) as indicated. An MMP inhibitor (iMMP) was administered on days 0-2. iMMP1 (Actinonin, a broad spectrum MMP inhibitor) and iMMP2 (Way-170523, a selective inhibitor of MMP-13). (**D**) Mouse weight over time. Data shown are mean+/-SEM. Ordinary one way ANOVA with Dunnett's multiple comparisons test was performed on weight at Day 4. *n*=1 experimental replicate.

Supplementary Figure 6

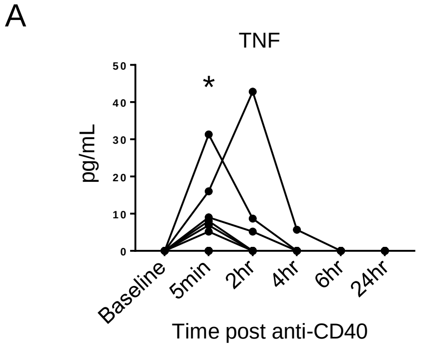

B

	Enriched Gene Sets (FDR q value <0.25)
1	HALLMARK_ALLOGRAFT_REJECTION
2	HALLMARK_INTERFERON_GAMMA_RESPONSE
3	HALLMARK_TNFA_SIGNALING_VIA_NFKB
4	HALLMARK_IL6_JAK_STAT3_SIGNALING
5	HALLMARK_INFLAMMATORY_RESPONSE
6	HALLMARK_G2M_CHECKPOINT
7	HALLMARK_INTERFERON_ALPHA RESPONSE
8	HALLMARK_E2F_TARGETS
9	HALLMARK_MITOTIC SPINDLE
10	HALLMARK_KRAS_SIGNALING_UP
11	HALLMARK_IL2_STAT5_SIGNALING
12	HALLMARK_COMPLEMENT
13	HALLMARK_EPITHELIAL_MESENCHYMAL_ TRANSITION
14	HALLMARK_ANGIOGENESIS
15	HALLMARK_APOPTOSIS
16	HALLMARK_HYPOXIA
17	HALLMARK_P53_PATHWAY
18	HALLMARK_APICAL_JUNCTION
19	HALLMARK_ESTROGEN_RESPONSE_LATE
20	HALLMARK_ESTROGEN_RESPONSE_EARLY
21	HALLMARK_APICAL_SURFACE
22	HALLMARK_COAGULATION
23	HALLMARK_TGF_BETA_SIGNALING
24	HALLMARK_HEDGEHOG_SIGNALING
25	HALLMARK_UNFOLDED_PROTEIN_RESPONSE
26	HALLMARK_MYC_TARGETS_V2

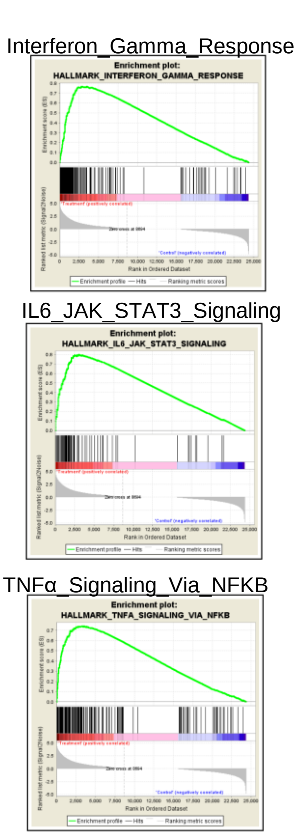

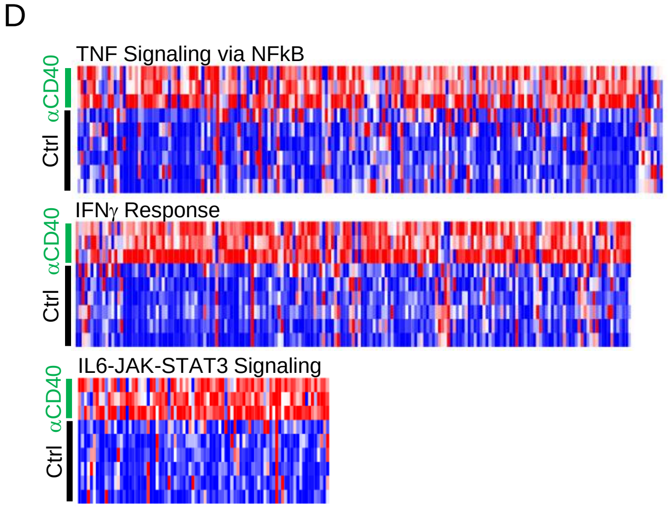

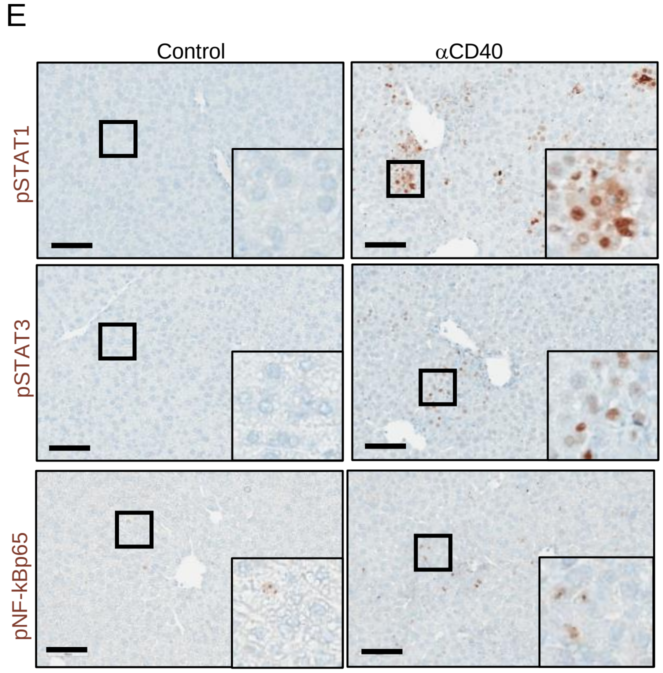

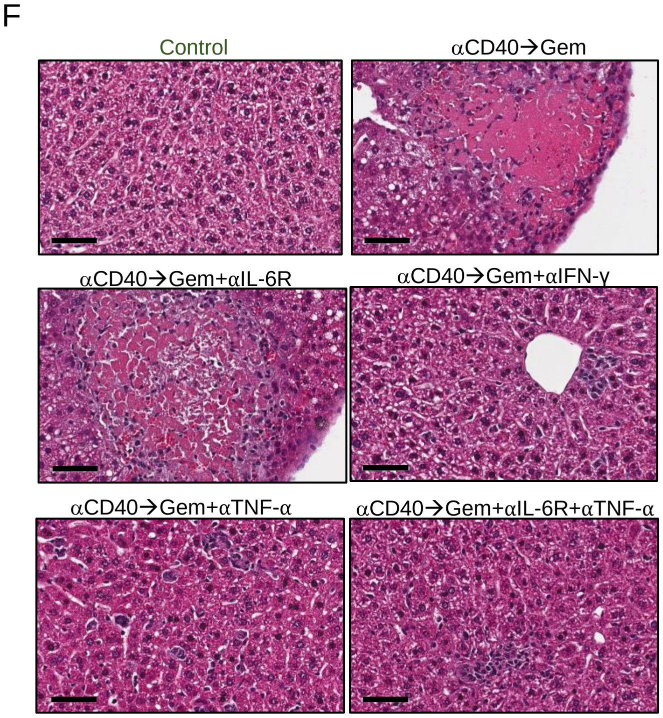

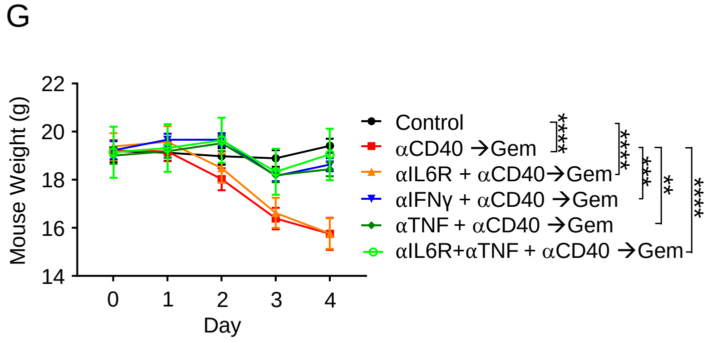

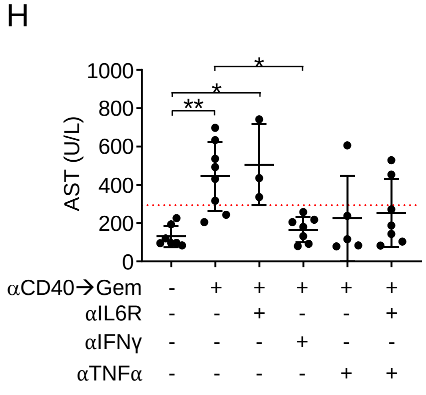

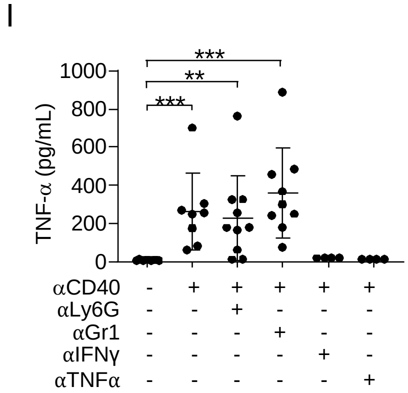

Supplementary Figure 6. CD40 agonist induces activation of IL-6, TNF, and IFN-γ signaling pathways in the liver. (A) TNF serum levels in patients (described in Figure 1A) after anti-CD40 treatment. Paired *t* tests were performed compared to baseline. *, $p<0.05$ **(B-D)** RNA was extracted from bulk liver tissue collected from mice at day 2 after treatment with control or FGK45 (αCD40). *n*=3-6 mice per group; 1 experimental replicate. Gene set enrichment analysis (GSEA) was performed. (**B**) Gene sets enriched with an FDR q value <0.25. **(C)** GSEA enrichment plots for indicated gene sets. (**D**) Heatmap of positively enriched genes in indicated gene sets. (**E**) Representative images showing detection of phosphorylated (p)STAT1, pSTAT3, and pNF-kBp65 by immunohistochemistry in liver tissues collected 2 days after treatment with αCD40 compared to control. Scale bars=100μm. *n*=8 mice/group; 1 experimental replicate. For **F-H**, mice were treated as described in Figure 3C. (**F**) Representative H&E images of necrotic lesions detected in the liver. Dashed lines indicate necrotic lesions. Scale bars = 60μm. **(G)** Mouse weight over time. Ordinary one-way ANOVA with Dunnett's multiple comparisons test was performed on weight at day 4. Comparisons to control and αCD40→Gem are shown. Data shown are mean+/-SEM. (**H**) AST serum levels at necropsy. Red line indicates upper range of 95% confidence interval for normal serum level of AST derived from all experiments in the manuscript. Data shown are mean+/-SD. Data for F-H are representative of *n*=2 experimental replicates. **(I)** Mice were treated with PBS or anti-Ly6G, anti-Gr1, anti-IFN-γ, or anti-TNF on Day -1. On Day 0, mice received anti-CD40 or isotype control, and anti-Gr1. Serum was collected at 18 hours later and analyzed by cytometric bead array for TNF. n=1 experimental replicate. **H, I)** Kruskal-Wallis with Dunn's multiple comparisons tests were performed. Comparisons between to control and αCD40 are shown..

Supplementary Figure 7

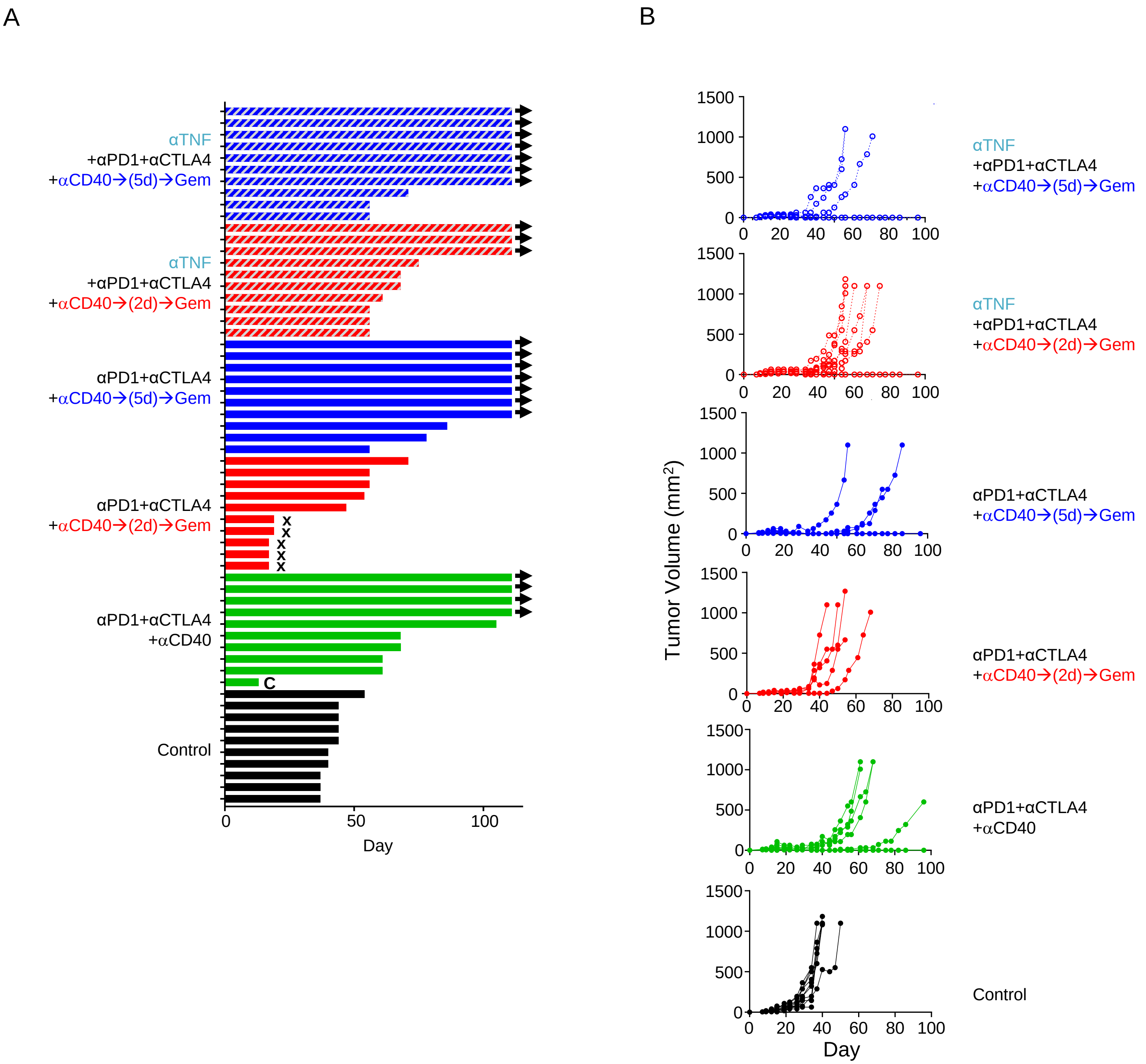

Supplementary Figure 7. TNF is dispensable for treatment efficacy mediated by CD40-based chemoimmunotherapy. Mice were treated as described in Figure 4. (**A**) Swimmer plot showing survival of individual mice in the indicated treatment groups. X, death from toxicity; C, censored; Arrows, indicate ongoing survival and cure. All other mice were euthanized for tumor size. (**B**) Individual mouse tumor growth over time. Data are representative of *n*=2 experimental replicates.
